# Is Caperatic Acid the Only Compound Responsible for Activity of Lichen *Platismatia glauca* within the Nervous System?

**DOI:** 10.3390/antiox11102069

**Published:** 2022-10-20

**Authors:** Elżbieta Studzińska-Sroka, Aleksandra Majchrzak-Celińska, Monika Bańdurska, Natalia Rosiak, Dominik Szwajgier, Ewa Baranowska-Wójcik, Marcin Szymański, Wojciech Gruszka, Judyta Cielecka-Piontek

**Affiliations:** 1 Department of Pharmacognosy, Poznan University of Medical Sciences, Rokietnicka 3 Str., 60-806 Poznań, Poland; 2 Department of Pharmaceutical Biochemistry, Poznan University of Medical Sciences, Święcickiego 4 Str., 60-781 Poznań, Poland; 3 Department of Biotechnology, Microbiology and Human Nutrition, University of Life Sciences in Lublin, Skromna 8 Str., 20-704 Lublin, Poland; 4 Centre for Advanced Technologies, Adam Mickiewicz University in Poznań, ul. Uniwersytetu Poznańskiego 10, 61-614 Poznań, Poland; 5 Department of Biological Sciences, Faculty of Physical Culture in Gorzów Wlkp., Poznan University School of Physical Education, Estkowskiego 13, 66-400 Gorzów Wielkopolski, Poland

**Keywords:** lichen extracts, biological activity, anti-neurodegenerative potential, anticancer activity

## Abstract

Lichens are a source of various biologically active compounds. However, the knowledge about them is still scarce, and their use in medicine is limited. This study aimed to investigate the therapeutic potential of the lichen *Platismatia glauca* and its major metabolite caperatic acid in regard to their potential application in the treatment of central nervous system diseases, especially neurodegenerative diseases and brain tumours, such as glioblastoma. First, we performed the phytochemical analysis of the tested *P. glauca* extracts based on FT-IR derivative spectroscopic and gas chromatographic results. Next the antioxidant properties were determined, and moderate anti-radical activity, strong chelating properties of Cu^2+^ and Fe^2+^ ions, and a mild effect on the antioxidant enzymes of the tested extracts and caperatic acid were proved. Subsequently, the influence of the tested extracts and caperatic acid on cholinergic transmission was determined by in vitro and in silico studies confirming that inhibitory effect on butyrylcholinesterase is stronger than against acetylcholinesterase. We also confirmed the anti-inflammatory properties of *P. glauca* extracts and caperatic acid using a COX-2 and hyaluronidase inhibition models. Moreover, our studies show the cytotoxic and pro-apoptotic activity of the *P. glauca* extracts against T98G and U-138 MG glioblastoma multiforme cell lines. In conclusion, it is possible to assume that *P. glauca* extracts and especially caperatic acid can be regarded as the source of the valuable substances to finding new therapies of central nervous system diseases.

## 1. Introduction

Neurodegenerative disorders and brain tumours are increasingly recognised as major causes of death and disability worldwide. A systematic analysis for the Global Burden of Disease Study 2016 revealed that the neurological disorders, including Alzheimer’s disease (AD) and other dementias as well as brain and other CNS cancers, were the leading cause of disability-adjusted life-years and second leading cause of deaths [1].

AD and other dementias are rising rapidly and will more than double in mortality burden over the next 20 years [2]. The major causes of the degeneration of neuronal cells include increase in oxidase enzymes’ activities, altered redox signalling and generation of reactive oxygen species. Other biochemical alterations responsible for neurodegeneration include abnormal protein folding, mitochondrial dysfunctions, and neuroinflammation [3]. Moreover, neurotransmitter disturbances are one of the essential dysfunctions observed in Alzheimer’s and non-Alzheimer’s dementias [4]. Nevertheless, the pathogenesis of AD is not fully understood and there is still no cure or even a fully functional treatment to slow the progression of the disease. However, the upliftment of cellular defence mechanisms and reducing the neuroinflammation are regarded as crucial in the treatment of Alzheimer’s and other neurodegenerative diseases [3]. Antioxidants, anti-inflammatory compounds or cholinesterase inhibitors, especially those derived from natural sources, can play an important role in delaying the onset as well as reducing the progression of AD [5].

Glioblastoma multiforme (GBM) is another important medical problem that accounts for a large and increasing health burden worldwide [6]. It is regarded as the most detrimental and aggressive brain neoplasm [7]. While GBM commonly shows dense proliferation of highly atypical and pleomorphic cells, necrosis and microvascular proliferation, its morphology and genetic background greatly varies from case to case [8]. The heterogeneous nature of GBM makes the development of an effective therapeutic approach extremely difficult. Currently, the standard GBM treatment involves surgery, radiotherapy and chemotherapy; however, the median survival time is estimated to be only approximately 14.6–16.6 months. Thus, novel treatment options are constantly sought.

The use of natural bioactive molecules is proposed as an alternative approach to the treatment and control of both neurodegenerative diseases and brain tumours [9]. It was reported that natural compounds obtained from fruits and vegetables, e.g., polyphenols, flavonoids, stilbenes, carotenoids and acetogenins, might be effective against cancer cells and might also protect from neurodegeneration [10,11]. Phytocompounds can produce anti-cancer effects through various mechanisms, including upregulation of apoptosis and autophagy, inducing cell cycle arrest, interfering with altered tumour metabolism and inhibiting proliferation and neuroinflammation [12]. Interestingly, growing body of evidence indicates that the natural compounds with anti-cancer properties are safe for normal neurons, or even show neuroprotective properties. The major mechanisms identified, through which phytochemicals exert their neuroprotective effects include antioxidant, anti-inflammatory, acetylcholinesterase and monoamine oxidase inhibiting properties [10].

Lichens are a group of organisms with a significant pharmacological potential. Growing body of evidence indicates that they are a promising source of unique and functional small molecules suitable for the treatment of CNS disorders, including neurodegenerative diseases and brain tumours. In this regard, cholinesterase’s inhibitory potential of lichen-derived compounds and extracts was reported [13,14]. Tyrosinase, the enzyme involved in the development of Parkinson’s disease, could be also blocked by lichen substances [14]. The anti-inflammatory effect was also detected, both for lichen-derived compounds and extracts [13,14]. Other studies indicate that lichen compounds can generate oxidative stress, interfere with the cell cycle distribution, induce apoptosis and inhibit the Wnt pathway, ameliorating the response of GBM cells to temozolomide (TMZ) [15].

**Figure 1:**
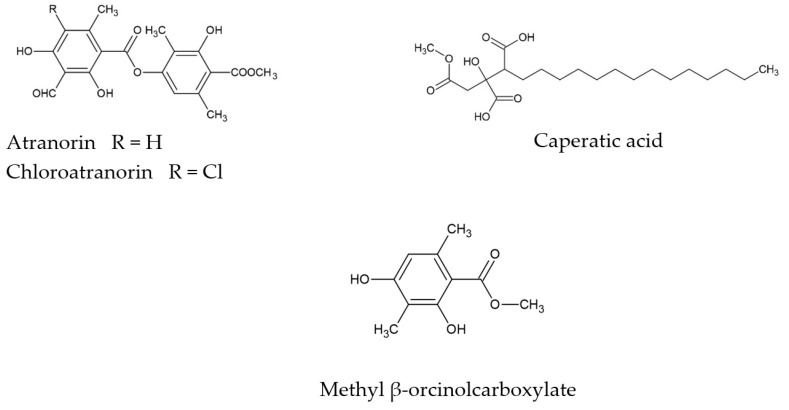
The chemical structures of the main secondary metabolites of *P. glauca*.

*Platismatia glauca* is a lichen from the *Parmeliaceae* family. Despite its common occurrence in many geographical regions of almost all continents, its biological potential is still poorly understood. The literature shows that one of the active metabolites present in the lichen thallus is caperatic acid [16]. Moreover, atranorin, chloroatranorin and methyl β-orcinolcarboxylate (atraric acid) were also identified [16,17]. Other authors also indicate pseudoplacodiolic acid and jackinic acid presence in the apothecias of this species [18]. The chemical structures of the main secondary metabolites of *P. glauca* are presented in Figure 1.

Until now, only a few studies were carried out concerning the biological activity of *P. glauca*. In this regard, the antimicrobial properties of *P. glauca* extracts were described [16,19,20]. The results of its antioxidant action [16,20] and the anti-cancer potential, were also demonstrated, especially in colon cancer cell lines [17,21]. Older studies also indicate a strong activity of lipophilic *P. glauca* extracts against Lewis lung cancer (3LL), leukaemia (L1210) cells [22] and glioblastoma (U251) cells [22]. Importantly, *P. glauca* extracts did not negatively influence the survival of various normal cells [17]. Our previous studies showed the anti-cancer potential of caperatic acid. In this regard, we reported the ability of caperatic acid to modulate β-catenin-dependent transcription resulting in cancer-specific cytotoxic effects in colorectal cancer cells and GBM cells [15,23]. In addition, we found that caperatic acid passes the blood–brain barrier via passive transport, which is a fundamental requirement for any CNS-targeting drug [15].

Until now, only a few studies were carried out concerning the biological activity of *P. glauca*. In this regard, the antimicrobial properties of *P. glauca* extracts were described [16,19,20]. The results of its antioxidant action [16,20] and the anti-cancer potential, were also demonstrated, especially in colon cancer cell lines [17,21]. Older studies also indicate a strong activity of lipophilic *P. glauca* extracts against Lewis lung cancer (3LL), leukaemia (L1210) cells [22] and glioblastoma (U251) cells [22]. Importantly, *P. glauca* extracts did not negatively influence the survival of various normal cells [17]. Our previous studies showed the anti-cancer potential of caperatic acid. In this regard, we reported the ability of caperatic acid to modulate β-catenin-dependent transcription resulting in cancer-specific cytotoxic effects in colorectal cancer cells and GBM cells [15,23]. In addition, we found that caperatic acid passes the blood–brain barrier via passive transport, which is a fundamental requirement for any CNS-targeting drug [15].

These promising results prompted us to continue the research on *P. glauca* extracts and caperatic acid. Thus, in this study the therapeutic potential towards the neurodegenerative disease and anti-GBM activity of dichloromethane (DCM), acetone (Ace), methanol (MeOH), methanol-water (MeOH-H_2_O) and water (H_2_O) extracts of *P. glauca* was evaluated and compared with the properties of *P. glauca* major secondary metabolite, caperatic acid.

## 2. Materials and Methods

### 2.1. Plant Material

The lichen *P. glauca* was manually collected from the bark of silver birch *Betula pendula* Roth in forest areas in the Piła commune (53°8′41.10″ N; 16°42′1.97″ E), Greater Poland in October 2018 and identified by Dr. Wojciech Gruszka (AWF in Poznań, Faculty in Gorzów Wlkp., Department of Biological Sciences). A voucher specimen (ES 2018.10) has been deposited in the herbarium of the Department of Pharmacognosy at the Poznan University of Medical Sciences.

### 2.2. Chemicals and Solvents

Sodium carbonate, sodium hydroxide, DMSO, acetone, dichloromrthane, methanol, ammonium acetate, copper (II) chloride were purchased from Avantor Performance Materials Poland S.A. (Gliwice, Poland). The Folin-Ciocalteu′s phenol reagent was from Merck (Darmstadt, Germany). Caperatic acid were isolated and identified in Department of Pharmacognosy of Poznan University of Medical Sciences [23] (Table S1), atranorin and methyl β-orcinolcarboxylate (atraric acid) were purchased from ChromaDex (Los Angeles, CA, USA). All other chemicals were from the Sigma–Aldrich Chemical Co. (Taufkirchen, Germany).

### 2.3. Preparation of Extract

The thallus of the *P. glauca* was dried at room temperature, cleaned of the bark, remaining impurities and crushed. Then 5.0 g of thallus was weighed for dichloromethane (DCM) and acetone (Ace) extracts or 4.0 g for the methanol (MeOH), methanol-water (MeOH-H_2_O) (1:1) or water (H_2_O) extracts and sonicated at 35 °C for 4 × 30 min with appropriate solvent (100 mL × 4) in an ultrasonic bath.

The extracts were filtered using Whatman filter paper No. 1 and concentrated by evaporation using a rotary evaporator (35–40 °C for dichloromethane and acetone, 55–60 °C for MeOH, MeOH–H_2_O (1:1) or H_2_O). The extracts were obtained with the yield: 3.89%, 5.21%, 9.72%, 7.54%, 6.76%, for DCM, Ace, MeOH, MeOH-H_2_O, and H_2_O extracts, respectively.

### 2.4. FT-IR Analyses of the Crude Extract

The ATR-FTIR spectra were collected on an IRTracer-100 spectrophotometer. All spectra were measured between 4000 and 400 cm^−1^ in the absorbance mode. The following spectrometer parameters were used: resolution: 4 cm^−1^, the number of scans: 400, and apodization: Happ–Genzel. The sample was placed directly on the ATR crystal. Solid samples were pressed against the ATR crystal and the ATR-FTIR spectrum was scanned. LabSolutions IR software (version 1.86 SP2, Shimadzu, Kyoto, Japan) was used to calculate the second derivative of all spectra. The software performed calculations according to the Savitzky–Golay numerical algorithm. The smoothing parameter was 25 points. A derivative spectrum allowed us to identify the peak positions of the original spectrum and to separate multiple peaks, which were adjoining or on the shoulder. The minima of the second derivative corresponded to the extremes of the original ATR-FTIR spectrum. Origin 2021b (OriginLab Corporation, Northampton, MA, USA) was used to analyse the acquired data.

### 2.5. GC-MS Analysis of the P. glauca Extracts

Chromatographic studies were performed on a GC-MS chromatograph (SCION TQ, BRUKER). About 10.0 µL of the sample was dissolved in 2.0 mL of CH_2_Cl_2_ (Sigma-Aldrich) and 1.0 µL of the solution was injected onto the column. The chromatograph was equipped with a VF-5ms Crawford Scientific silica column. The electron energy was 70 eV, and the ion source was at 200 °C. Helium was used as the carrier gas at a flow rate 1.0 mL/min. Temperature program: Enable Coolant at 50.0 °C, Coolant Timeout 20.00 min, Stabilization Time 0.50 min; Temperature 60.0 °C, Hold 3.00 min., Total 3.00 min.; Temperature 280.0 °C, Rate 10.0 °C/min., Hold 35.00 min., Total 60.00 min. The identification of compounds was based on a comparison of their retention time as well as mass spectra with NIST standards.

### 2.6. Total Phenolic Content (TPC)

The analysis was carried out according to Studzińska-Sroka et al. [13]. The extracts were prepared for the study at 2 mg/mL. Briefly, 25.0 µL of the examined extract or standard, 200.0 µL of distilled water, 15.0 µL of Folin-Ciocateau reagent, and 60.0 µL of 20% calcium carbonate solution were added sequentially to the wells. As the blank, the mixture of the reagents without the tested extract/reference compound were used. The plate was shaken at 600 rpm for 5 min. (at room temperature and in the dark) and after that, the incubation for 25 min was continued. The absorbance was measured using 760 nm as the wavelength (spectrophotometer UV/VIS, Lambda 35, Elmer–Perkin). The samples were run in triplicate and the results were presented as mg of gallic acid equivalent (GAE) per g of a dry extract ± standard deviation (SD).

### 2.7. Antioxidant Activity

#### 2.7.1. ABTS and CUPRAC Analysis

Two methods were used to test the antioxidant activity: ABTS and CUPRAC approach. The ABTS assay was effected according to Chanaj-Kaczmarek et al. (2015) [24] with slight modifications. Briefly, 10.0 μL of examined extracts or references substances, prepared at different concentrations (the range 0.5–8.0 mg/mL, for each extracts; the range 0.01–0.07 mg/mL, for quercetin; the range 0.0125–0.2 mg/mL, for vitamin C) were mixed with 200.0 μL of ABTS^•+^ solution (7 mM ABTS^•+^ prepared in 2.45 mM potassium persulfate solution and diluted with water to absorbance value ~0.77). The reached final assay concentrations were: 24–381 μg/mL, for extracts; 0.48–3.3 μg/mL, for quercetin; 0.6–9.52 μg/mL, for vitamin C). Afterward, the samples were incubated in the dark (30.00 min, shakingat 500 rpm) at room temperature. Next, the absorbance was measured at 734 nm. The control contained 10.0 μL of DMSO and 200.0 μL of ABTS^•+^ solution. The blanks for samples and control were the mixture of 10 μL of extract or 10.0 μL of DMSO, respectively and 200.0 μL of distillate water. For calculating the scavenging % of ABTS^•+^, the following formula was used:

ABTS^•+^ activity [%] = [(A_c_ − A_s_)/A_c_] × 100% (1)

where A_c_ is the absorbance of the control and A_s_ is the absorbance of the sample. 

The IC_50_ values, i.e., (a concentration of antioxidant necessary to half of the initial ABTS^•+^ quantity), were used to compare the quality of the antioxidant potency of the studied extracts. The lower absorbance of the reaction mixture indicated a higher ROS scavenging activity. For the investigated substances, two independent experiments were carried out and the average from *n* = 6 measurements was calculated.

For measuring the antioxidant capacity of the lichen extracts and compounds the CUPRAC assay was used [14]. Composed of equal parts of acetate buffer (pH 7.0), 7.5 mM neocuproine solution in 96% ethanol, and 10 mM CuCl_2_·H_2_O solution, the CUPRAC reagent was prepared immediately before the analysis. The samples (50.0 μL) dissolved in DMSO at different concentrations (62.5–2000 μg/mL, for extracts, and 250–4000 μg/mL for caperatic acid), was mixed with the CUPRAC reagent (150.0 μL) (the final assay concentrations were: 15.6–500 μg/mL, for extracts, and 62.5–1000 μg/mL, for caperatic acid). Next, the samples were shaken and incubated in the dark at room temperature for 30 min. The absorbance measure was performed at 450 nm. Vitamin C was used as a reference (12.5–200 μg/mL; the final assay concentrations were 3.1–50 μg/mL).

The results were expressed as the IC_0.5_, the concentration at which the absorbance was 0.5. For the investigated substances, two independent experiments were carried out, and the average from *n* = 6 for extracts, and *n* = 3 for caperatic acid, measurements were calculated.

#### 2.7.2. Chelating Activity of Fe^2+^ and Cu^2+^

Two methods were used to test the chelating ability of the extracts from *P. glauca*.First we examine the Fe^2+^ chelation ability. The study was conducted according to the methodology described by Studzińska-Sroka et al. (2021) [25] with some modifications. Briefly, 200.0 µL of tested extracts (the concentration range 0.2–5.0 mg/mL) or the reference substance (the concentration range 0.2–5.0 mg/mL), dissolved in the methanol-DMSO (1:1), and 10.0 µL of iron (II) chloride aqueous solution were mixed (the final assay concentrations were: 0.16–4.0 mg/mL). Next, the plate was shaken (500 rpm) at room temperature for 10 min. Then, 40.0 µL of ferrozine aqueous solution was added. The plate was reincubated for 10 min at room temperature with shaking (500 rpm). The absorbance was measured at λ = 562 nm. The control sample contained 200.0 µL of the methanol-DMSO (1:1) mixture and 10.0 µL of iron (II) aqueous chloride solution. The blanks for extracts and reference were prepared without the ferrozine replacing it with distilled water. The chelating capacity of iron (II) ions was calculated using the equation:

(2) $${\mathrm{Fe}}^{2+}\mathrm{chelating}\;\mathrm{activity}\;\left[\%\right]=1-\frac{\left(\mathrm{As}\;-\mathrm{Abs}\right)}{\left(\mathrm{Ac}-\mathrm{Abc}\right)}\times 100$$

where As is the absorbance of the sample, Abs is the absorbance of the blank of the sample, Ac is the absorbance of the control and Abc is the absorbance of the blank of the control. For the investigated substances, two independent experiments were carried out, and the average from *n* = 4 for extracts, and *n* = 6 for the reference, measurements were calculated.

Whereas the ability extracts to chelate Cu^2+^ was assessed using the colorimetric method described by Santos et al. (2017) [26], with some modifications. Briefly, 30.0 µL of the examined extract or positive control (DMSO diluted) was pipetted to the 96-wells plate. Subsequently, 175.0 µL of sodium acetate buffer (50 mmol/L, pH 6.0) and a 30.0 µL of a 100 mg/L CuSO_4_·5H_2_O solution were added. Then the plate was shaken for 10 min (300 RPM) at room temperature. After this time the samples were filled with 15.0 µL of 2 mM pyrocatechol violet, and the 20 min incubation (room temperature) was effectuated. The absorbance was measured at 632 nm using microplate reader. The concentration of lichen extract was from 0.041 mg/mL (DCM and Ace or quercetin) to 0.66 mg/mL or 0.083 mg/mL to 1.66 mg/mL (MeOH, MeOH–H_2_O, H_2_O)

(3) $${\mathrm{Cu}}^{2+}\mathrm{chelating}\;\mathrm{activity}\;\left[\%\right]=1-\frac{\left(\mathrm{As}-\mathrm{Abs}\right)}{\left(\mathrm{Ac}-\mathrm{Abc}\right)}\times 100$$

where As is the absorbance of the sample, Abs is the absorbance of the blank of the sample, Ac is the absorbance of the control and Abc is the absorbance of the blank of the control. For the investigated substances, two independent experiments were carried out, and the average from *n* = 4 measurements was calculated.

#### 2.7.3. Effect on Antioxidant Enzymes Activity

Samples of the *P. glauca* extracts and caperatic acid were dissolved in DMSO to obtain the concentration of 2 mg/mL.

##### Effect on Catalase Activity (CAT)

Sample (20.0 µL) was mixed in a plate with 100.0 µL of EDTA solution (16.5 mg/mL of Tris-HCl buffer, pH 7.0, 6.06 g/50 mL), 20.0 µL H_2_O_2_ working solution (10.0 µL of 30% H_2_O_2_ solution diluted to 10 mL by the buffer), and 10.0 µL of catalase Sigma-Aldrich C3515, initially 200-fold diluted in buffer). The volume was completed by 14.0 µL of the same Tris-HCl buffer in order to mix the sample, followed by the incubation at 20 °C for 30 min [27]. Then, 152.0 µL ABTS/peroxidase solution and 76.0 µL of HCl working solutions were added followed by direct absorbance measurement at 414 nm. ABTS/peroxidase working solution was prepared as follows: 1.98 mg ABTS (Sigma A1888) and 2 mg horseradish peroxidase (Sigma 516531) were dissolved in Tris-HCl buffer (pH 7.0, 6.06 g/50 mL) to obtain 15 mL of the reagent. HCl working solution was obtained by mixing 628.9 µL HCl (Sigma 258148) with distilled water to obtain 10 mL [28]. Inhibition [%] was calculated as the difference in the increase of absorbance (414 nm) between the tested sample and the control that contained buffer instead of the studied sample (taken as 100%). Backgrounds of samples were measured by mixing 20.0 µL of the sample completed with Tris-HCl to obtain the final reaction volume.

##### Effect on Superoxide Dismutase Activity (SOD)

Sample (50.0 μL) was mixed with 10.0 μL SOD (0.24 U), 160.0 μL nitrobluetatrazolium solution (0.0025 M), 140.0 μL phosphate buffer (0.2 M, pH 7.5), 30.0 μL xanthine (150 mM in 1 M NaOH) and 10.0 μL xanthine oxidase (0.065 U, Sigma Aldrich, St. Louis, MO, USA, X4875). Every 1 min, the change in the absorbance at 550 nm was measured in tested samples vs. controls without the studied sample and the effect on the enzyme was calculated using equation [29]:

(4) $$\mathrm{SOD}\;\mathrm{inhibition}\;\left[\%\right]=100-100\times \frac{\left(\mathrm{Abs}.15\;\min\;-\;\mathrm{Abs}.0\;\min\right)}{\left(\mathrm{Abs}.\;\mathrm{control}.15\;\min-\;\mathrm{Abs}.\;\mathrm{control}.0\;\min\right)}$$

##### Effect on Glutathione Reductase (GR) Activity

Sample (150.0 μL) was mixed with 660.0 μL of 0.1 mM sodium phosphate buffer, 25 μL of EDTA solution and 30.0 μL of GSSG solution, incubated for 5 min. at 25 °C, then 40 μL of NADPH solution and was added (all reagents were dissolved in 0.1 mM sodium phosphate buffer, pH 7.6), and the initial absorbance (340 nm) was recorded. Then, the reaction was started by addition of 2 U enzyme (2.0 μL, Sigma Aldrich no G3664), and the absorbance was recorded after 10 min of incubation at 25 °C. Concentrations of reagents in the final mixture (805.0 μL) were as follows: 0.5 mM EDTA, 10 mM GSSG and 10 mM NADPH. Blank sample was prepared with buffer instead of the sample and background was measured (mixture containing studied sample and buffer only). The effect on the enzyme was calculated using equation [30]:

(5) $$\mathrm{GR}\;\mathrm{inhibition}\;\left[\%\right]=100-100\times \frac{\left(\mathrm{Abs}.10\;\min\;-\;\mathrm{Abs}.0\;\min\right)}{\left(\mathrm{Abs}.\;\mathrm{control}.10\;\min\;-\;\mathrm{Abs}.\;\mathrm{control}.0\;\min\right)}$$

##### Effect on Glutathione Peroxidase (GPx) Activity

Sample (150.0 μL) was mixed with 20.0 μL of EDTA solution, 3.0 μL of glutathione reductase (0.2 U Sigma G3664), 10.0 μL of GSH solution, 3 μL of glutathione peroxidase (0.04 U Sigma G6137), 55.0 μL of H_2_O_2_ and 663.0 μL of 50 mM sodium phosphate, pH 7.0). To start the reaction, 10 μL of NADPH solution (N5130) was added and the decrease in the absorbance (340 nm) was read after 5 min of incubation at 25 °C. All solutions were prepared in 50 mM buffer and concentrations of reagents in the final mixture were as follows: 1 mM EDTA, 0.2 U glutathione reductase, 2 mM GSH, 0.04 U glutathione peroxidase, 1.5 mM H_2_O_2_ and 0.8 mM NADPH. Blank sample was prepared with buffer instead of the sample and background was measured (mixture containing studied sample and buffer only). The effect on the enzyme was calculated using equation [31]:

(6) $$\mathrm{GPx}\;\mathrm{inhibition}\;\left[\%\right]=100-100\times \frac{\left(\mathrm{Abs}.5\;\min\;-\;\mathrm{Abs}.0\;\min\right)}{\left(\mathrm{Abs}.\;\mathrm{control}.5\;\min\;-\;\mathrm{Abs}.\;\mathrm{control}.0\;\min\right)}$$

### 2.8. Anticholinesterase Activity

#### 2.8.1. Effect on Acetylcholinesterase (AChE) Activity

Inhibition of acetylcholinesterase (AChE) was determined by a method described by Studzińska-Sroka et al. [13] with some modifications. Briefly, to the 5.0 µL of *P. glauca* extracts and caperatic acid (dissolved in DMSO at 10 mg/mL and 40 mg/mL; final concentration 0.2 mg/mL and 0.8 mg/mL), the 60.0 µL of TRIS-HCl buffer (50 mM, pH = 8) was added and 30 µL of AChE (0.2 U/mL). After that, the plate was incubated for 5 min at 25 °C with shaking (500 rpm). Next 30.0 µL of acetylthiocholine iodide (1.5 mM) and 125.0 µL of 5,5′-dithiobis(2-nitrobenzoic acid (0.3 mM with 10 mM NaCl and 2 mM MgCl_2_·6H_2_O) were added. After the 30 min incubation at 25 °C with shaking (500 rpm), the absorbance was measured at 405 nm (Multiskan GO 1510, Thermo Fisher Scientific, Vantaa, Finland). The galantamine was used as reference (dissolved in DMSO at 0.078–5 mg/mL; final concentration 0.004–0.25 mg/mL). All experiments were carried out three times, and the average from *n* = 6 measurements was calculated. The control sample contained 5.0 µL of DMSO instead of the tested sample. The blank for control was prepared without AChE replacing it with TRIS-HCl buffer. The blanks for samples were prepared without AChE replacing it with TRIS-HCl buffer. The percentage of enzyme inhibition was calculated using the equation:

(7) $$\mathrm{AChE}\;\mathrm{inhibition}\;\left[\%\right]=1-\frac{\left(\mathrm{As}\;-\;\mathrm{Abs}\right)}{\left(\mathrm{Ac}\;-\;\mathrm{Abc}\right)}\times 100$$

where As is the absorbance of the sample, Abs is the absorbance of the blank of the sample, Ac is the absorbance of the control, and Abc is the blank of the control.

#### 2.8.2. Effect on Butyrylcholinesterase (BChE) Activity

Inhibition of butyrylcholinesterase (BChE) was determined by a method described by Studzińska-Sroka et al. [13] with some modifications. Briefly, to the 5.0 µL of *P. glauca* extracts and caperatic acid (dissolved in DMSO at 10 mg/mL and 40 mg/mL; final concentration 0.2 mg/mL and 0.8 mg/mL), 60.0 µL of TRIS-HCl buffer (50 mM, pH = 8) and 30.0 µL of BChE (0.2 U/mL) were added. After that, the plate was incubated for 5 min at 25 °C with shaking (500 rpm). Next 30.0 µL of butyrylthiocholine iodide (1.5 mM) and 125 µL of 5,5′-dithiobis(2-nitrobenzoic acid (0.3 mM with 10 mM NaCl and 2 mM MgCl_2_·6H_2_O) were added. After the 30 min incubation at 25 °C with shaking (500 rpm), the absorbance was measured at 405 nm (Multiskan GO 1510, Thermo Fisher Scientific, Vantaa, Finland). Galantamine was used as reference (dissolved in DMSO at 0.016–1 mg/mL; final concentration 0.0003–0.02 mg/mL). All experiments were carried out three times, and the average from *n* = 6 measurements was calculated. The control sample contained 5.0 µL of DMSO instead of the tested sample. The blank for control was prepared without BChE replacing it with TRIS-HCl buffer. The blanks for samples were prepared without BChE replacing it with TRIS-HCl buffer. The percentage of enzyme inhibition was calculated using the equation:

(8) $$\mathrm{BChE}\;\mathrm{inhibition}\;\left[\%\right]=1-\frac{\left(\mathrm{As}\;-\;\mathrm{Abs}\right)}{\left(\mathrm{Ac}\;-\;\mathrm{Abc}\right)}\times 100$$

where As is the absorbance of the sample, Abs is the absorbance of the blank of the sample, Ac is the absorbance of the control, and Abc is the blank of the control.

### 2.9. Molecular Docking on Cholinesterse Activity

Molecular docking studies were performed to investigate the binding mode between caperatic acid and the two enzymes (AChE and BChE) using MGLTools 1.5.6 with AutoDock 4.2 (ADT; Scripps Research Institute, La Jolla, San Diego, CA, USA) [32]. The three-dimensional (3D) structures of the AChE (AChE, PDB id: 4M0E) and butyrylcholinesterase (BChE, PDB id: 4BDS) were downloaded from RCSB Protein Data Bank [33]. The structure of caperatic acid (PubChem CID: 193112) was downloaded from PubChem [34].

The caperatic acid structure was optimized, using Gaussview program (Wallingford, CT, USA, Version E01), then added with hydrogen atoms and Gasteigr’s partial charge in AutoDock [32,35]. Next, the ligands and water molecules in the AChE and BChE structure were removed. Files were saved in PDB format. These files were imported into the Chimera 1.16 program [36] where the structure repair process was performed (e.g., added missing elements—hydrogen atoms and missing atoms) [37]. The structures were automatically saved in the MOL2 format, the file was converted to the PDB format with the Open Babel program [38,39]. The files prepared in this way were re-entered into the AutoDock Tools. The distance between the surface area of the enzymes and the caperatic acid molecule was limited to the maximum radius limit of 0.375 Å. All molecular docking simulations were demonstrated using a grid box measuring 126 × 126 × 126 (the search grid of the key site of AChE and BChE were identified as centre x:−0.693 y:−51.854 z: 3.057 and x: 138.789 y: 123.567 z: 38.56, respectively) and the Lamarckian Genetic Algorithm method with 100 conformations. After docking simulations, the best scoring pose was selected and exported to the PDBQT format. The file was converted to the PDB format with the Open Babel program and open in the Protein-Ligand Interaction Profiler [40,41] which was used to analyse the resulting interactions. Next download visualization in PyMol format and open in PyMol 2.5.1 (DeLano Scientific LLC, Palo Alto, CA, USA) to save the image The PyMOL Molecular Graphics System, Version 2.0 Schrödinger, LLC, New York, USA [42].

### 2.10. Effect on Cyclooxygenase-2 (COX-2) Activity

For the assay, reagents from Cayman COX Activity Assay Kit (No. 760151) were prepared strictly as suggested by the producer and were combined with COX-2 enzyme (Human recombinant, Cayman No. 60122, pre- diluted 100-fold using 100 mM, pH 8.0 Tris buffer). A volume of 40.0 μL of studied sample was mixed with 120.0 μL of Tris buffer (100 mM, pH 8.0), 10.0 μL hemin, shaken and left for 5 min at 25 °C followed by addition of 20.0 μL colorimetric substrate and 20.0 μL arachidonic acid solution. To start the reaction, 20.0 μL of COX-2 solution was added. The increase of the absorbance during the incubation at room temperature (20 min) was recorded at 590 nm. Negative (blank) sample (buffer instead of studied sample) and positive sample (COX-2 inhibitor DuP-697) were run simultaneously. Background of studied samples (40.0 μL of sample mixed with 190.0 μL buffer) was also measured and included in the calculations. Each sample was run in at least four repeats. Inhibition of the enzyme activity was expressed in % (indicates by how many % the activity has been reduced in relation to the negative -blank sample for which the maximum activity was assumed as 100%, under the conditions used in the method). Moreover, inhibition of enzyme activity was expressed as acetylsalicylic acid equivalent concentration (mg/mL). For this purpose, acetylsalicylic acid solutions were prepared at 14 concentrations (0.2–10 mg/mL), and analysed similarly to tested samples.

### 2.11. Anti-Hyaluronidase Activity

Inhibition of hyaluronidase was determined by a method described by Studzińska-Sroka et al. [14]. Briefly, to the tested well, 25.0 µL of incubation buffer (50 mM, pH 7.0, with 77 mM NaCl and 1 mg/mL of albumin) was added. Next, 25.0 µL of enzyme (30 U/mL of acetate buffer pH 7.0), 10.0 µL of the tested extracts or caperatic acid (0.156–1.25 mg/mL for dichloromethane, acetone, MeOH, MeOH:H_2_O extracts, and 1.25–2.5 mg/mL for H_2_O extract) or caperatic acid (0.078–0.625 mg/mL), and 15.0 µL of acetate buffer (pH 4.5) were mixed (the final concentrations were: 0.0156–0.125 mg/mL or 0.0078–0.0625 mg/mL, respectively). Subsequently the samples were incubated at 37 °C for 15 min., and after that 25.0 µL of hyaluronic acid (0.3 mg/mL in acetate buffer) was added. After the 45 min incubation (37 °C), 200.0 µL of 2.5% CTAB in 2% NaOH was added. The samples were incubated for 10 min (25 °C) and the turbidance was measured as the absorbance at 600 nm (Multiskan GO 1510, Thermo Fisher Scientific, Vantaa, Finland). The β-escin was used as the positive control (5.0–10.0 mg/mL, with the final concentration 0.5–1.0 mg/mL). All experiments were carried out three times and the average from *n* = 4 (lichen substance and extracts) or *n* = 5 (*β*-escin) measurements was calculated. The calculation of the percentage of inhibition was carried out using the equation below.

(9) $$\mathrm{Hyal}\;\mathrm{inhibition}\;[\%]=\frac{\left(T_{s}-{\mathrm{TE}}_{\mathrm{blank}}\right)}{\left({\mathrm{TH}}_{\mathrm{blank}}-{\mathrm{TE}}_{\mathrm{blank}}\right)}\times 100\%$$

where Hyal—hyaluronidase; T_S_—absorbance of sample; TE_blank_—absorbance of the enzyme + examined substance; TH_blank_—absorbance of the hyaluronic acid + examined substance.

### 2.12. Cell Culture and Media

T98G and U-138 MG were obtained from certified sources, namely European Collection of Authenticated Cell Cultures (ECACC) and American Type Culture Collection (ATCC), respectively. ATCC-formulated Eagle’s minimum essential medium (EMEM) (Merck, Germany) was used for both cell cultures. The medium was supplemented with FBS (Biowest, Nuaillé, France) to a final concentration of 10%, as well as antibiotics (penicillin and streptomycin) (Merck, Darmstadt, Germany) to the final concentrations of 1%. For the experiments, the amount of FBS was reduced to 5%. Additionally, the medium for T98G was supplemented with 2 mM glutamine, 1% non-essential amino acids, and 1% sodium pyruvate (all purchased from Merck, Germany). The cells were propagated at 37 °C and 5% CO_2_ in a humidified incubator (Memmert, Schwabach, Germany).

#### 2.12.1. In Vitro Cytotoxicity and Determination of IC_50_

The 50% inhibitory concentration (IC_50_) value was determined using 3-(4,5-dimethylthiazol-2-yl)-2,5- diphenyltetrazolium bromide (MTT) assay carried out according to our previously described method [43]. Briefly, 10,000 cells were seeded on 96-well plates and incubated for 24 h. Afterwards the medium was changed and the extracts were added in increasing concentrations (10, 25, 50 and 100 µM). The incubation time was 48 h. Then, the cells were washed with PBS, and MTT solution (200.0 µL of 0.5 mg/mL MTT dissolved in cell culture medium l) was added to each *P. glauca* treated and control wells. The plates were subsequently incubated for 4 h at 37 °C. Cell viability assay was performed by the conversion of the tetrazolium salt (MTT) to a purple colour formazan crystals by the mitochondrial dehydrogenases. Afterwards, the crystals were dissolved in 200.0 µL of acidic isopropanol and absorbance was measured at λ = 570 nm and λ = 690 nm on the microplate reader (Tecan Infinite M200, Grödig, Austria). All the experiments were repeated three times with four measurements per assay.

#### 2.12.2. Apoptosis Analysis

Apoptosis assay was performed with the Muse^®^ Cell Analyzer instrument, and its relative Muse^®^ Annexin V Dead Cell Kit (Merck, Darmstadt, Germany) according to the instructions supplied by the manufacturer. The kit utilises Annexin V and 7-aminoactinomycin D to detect early and late apoptotic cells. Annexin V detects phosphatidyl serine, an apoptotic marker that is exposed out of the external membrane of apoptotic cells, while 7-aminoactinomycin D is a DNA intercalating molecule used as an indicator of cell membrane integrity (it can bind DNA only in cells undergoing late apoptosis/death stage, when the membrane integrity is lost). Briefly, 10,000 cells were seeded on 6-well plates and incubated for 24 h. Afterwards, *P. glauca* extracts were added to the wells to the concentration of 50 µg/mL. The apoptosis analysis was performed after 48 h. Briefly, cells were washed with sterile 1× phosphate-buffered saline (PBS), trypsinized, resuspended, and diluted (1:1) with the Muse Annexin V Dead Cell reagent. The analysis was performed after 20 min of incubation.

#### 2.12.3. Cell Cycle Analysis

The assay performed with Muse™ Cell Cycle Kit and Muse™ Cell Analyzer (Merck, Darmstadt, Germany) identifies and measures the number of cells within the various phases (G0/G1, S and G2/M) of the cell cycle. The analysis was performed according to the manufacturer’s instructions. Briefly, after 48 h of incubation with the analysed compounds, the cells were trypsinized, washed with PBS buffer, fixed in ice-cold 70% ethanol, and stored until staining at −20 °C. Before the analysis, the fixed cells were washed with PBS buffer, stained, and subjected to 0.5 h incubation at room temperature in the darkness. Cells treated with the vehicle DMSO were used as negative, while cells treated with 100 nM and 500 nM topotecan served as positive controls. The subsequent analysis was performed on Muse™ Cell Analyzer (Merck, Germany).

### 2.13. Statistical Analysis

Routine statistical tests were performed using a one-way analysis of variance (ANOVA), and statistical differences (using Tukey’s HSD test or Duncan’s tests) with the significance threshold of *p* 0.05 were determined. All statistical analyses were performed using Statistica 13.1 software (StatSoft, Poland).

## 3. Results

### 3.1. Phytochemicals Analysis of P. glauca

#### 3.1.1. FT-IR Analyses

To confirm the presence of the major active compounds in *P. glauca* extracts, FT-IR analysis was performed. For more accurate identification, second derivative spectroscopy was employed to increase the apparent spectral resolution. Savitzky—Golay polynomial fitting method (LabSolution IR software, Shimadzu, Tokyo, Japan) was employed to enhance the separation of overlapping peaks [44,45,46].

**Figure 2:**
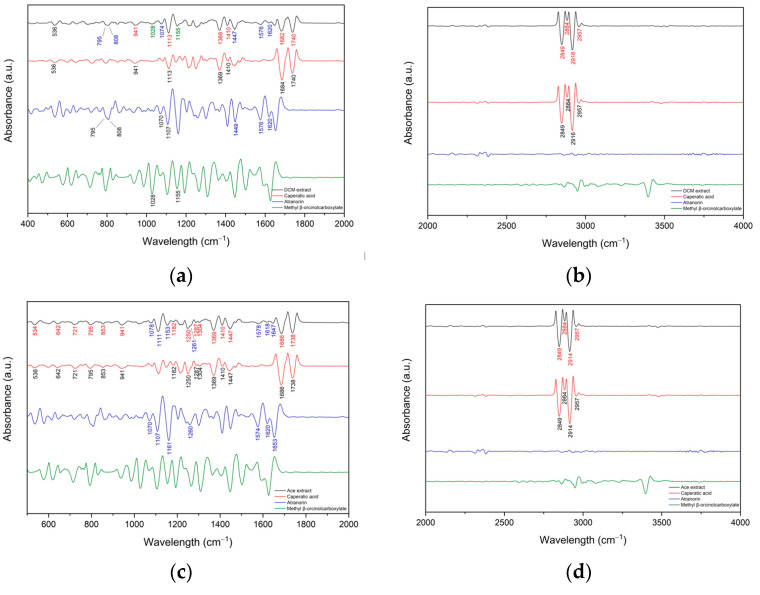

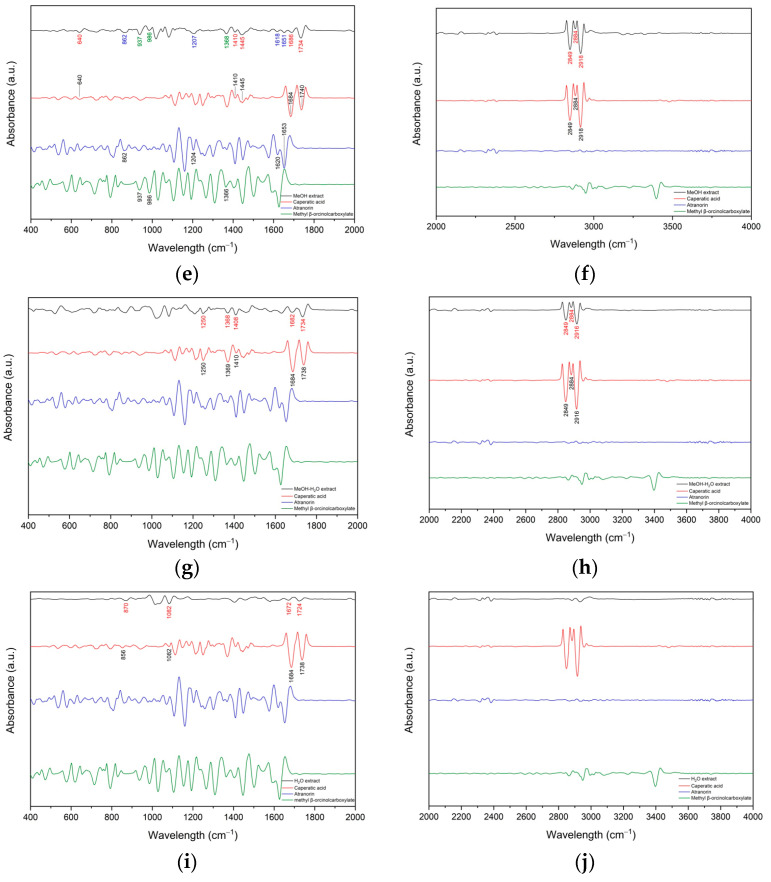
Second derivative infrared spectra (by the Savitzky–Golay polynomial fitting method, 25-point smoothing) of DCM extract range 400–2000 cm^−1^ (**a**), DCM extract range 2000–4000 cm^−1^ (**b**), Ace extract range 400–2000 cm^−1^ (**c**), Ace extract range 2000–4000 cm^−1^ (**d**), MeOH extract range 400–2000 cm^−1^ (**e**), MeOH extract range 2000–4000 cm^−1^ (**f**), MeOH-H_2_O extract range 400–2000 cm^−1^ (**g**), MeOH-H_2_O extract range 2000–4000 cm^−1^ (**h**), H_2_O extract range 400–2000 cm^−1^ (**i**), H_2_O extract range 2000–4000 cm^−1^ (**j**); (black line) juxtaposed with the spectra of caperatic acid (red line), atranorin (blue line) and methyl β-orcinolcarboxylate (green line).

Figure 2a–j shows the second derivative infrared spectra of the DCM, Ace, MeOH, MeOH-H_2_O, H_2_O extract and the standards (caperatic acid, atranorin and methyl β-orcinolcarboxylate). The second derivative spectra of the extracts possess more observable corresponding peaks and present similar absorption characteristics to standards. The most characteristic peaks are described in the supplementary material (Table S2). In the DCM extract bands corresponding to caperatic acid predominate (Figure 2a,b). Bands observed at about 795 cm^−1^, 808 cm^−1^, 1074 cm^−1^, 1576 cm^−1^ and 1620 cm^−1^ probably match atranorin while the bands at around 1028 cm^−1^ and 1155 cm^−1^ may be from trace amounts of methyl β-orcinolcarboxylate. Next in the Ace extract, the presence of caperatic acid and atranorin has been confirmed (Figure 2c,d). In the MeOH extract there are few bands that can be attributed to the presence of caperatic acid (640 cm^−1^, 1410 cm^−1^, 1445 cm^−1^, 1686 cm^−1^, 1734 cm^−1^, 2849 cm^−1^, 2884 cm^−1^ and 2918 cm^−1^), atranorin (862 cm^−1^, 1207 cm^−1^, 1618 cm^−1^ and 1651 cm^−1^) and methyl β-orcinolcarboxylate (937 cm^−1^, 986 cm^−1^ and 1368 cm^−1^) (Figure 2e,f). Bands observed in the spectrum of MeOH-H_2_O extract indicate the presence of caperatic acid (1250 cm^−1^, 1368 cm^−1^, 1408 cm^−1^, 1682 cm^−1^, 1734 cm^−1^, 2849 cm^−1^, 2884 cm^−1^, 2916 cm^−1^) (Figure 2 g,h). The least rich in bands is H_2_O extract (870 cm^−1^, 1082 cm^−1^, 1672 cm^−1^ and 1724 cm^−1^) (Figure 2i,j), therefore the presence of caperatic acid is uncertain.

#### 3.1.2. GC-MS Analysis of the *P. glauca* Extracts

The volatile compounds of *P. glauca* extracts were analysed using the GC–MS and the qualitative and quantitative compositions are presented in Table 1. The study was carried out with five examined extracts: DCM, Ace, MeOH, MeOH-H_2_O and H_2_O. About 42 different compounds were identified in *P. glauca* extracts including nineteen, nine, five, six, and six compounds in the DCM, Ace, MeOH, MeOH-H_2_O, and H_2_O extracts, respectively. The major components were 5-nonanone (60.01% in DCM extract), 2,4-dihydroxy-3,6-dimethylbenzoic acid, (46.50% in Ace extract), 2-buten-1-one, 1,3-diphenyl (36.41% in MeOH extract), 3-phenylbutyrophenone and 2,3,3-trimethyl-5-phenyl-1-pentene, (43.36% and 41.56% in MeOH-H_2_O extract) and triphenylphosphine, (56.81% in H_2_O extract).

#### 3.1.3. Total Polyphenols Content

The total polyphenols content was determined using the spectrophotometric Folin-Ciocalteau method. The tested extracts of *P. glauca* were characterized by a differential content of phenolic compounds. The highest quantity of polyphenols was measured for MeOH extract (39.11 ± 1.19 mg GAE/g of extract). A similar content was noted for Ace (36.78 ± 0.52 mg GAE/g of extract) and DCM (31.61 ± 0.49 mg GAE/g of extract) extracts. The more polar MeOH-H_2_O or H_2_O extracts were poorer in the polyphenolic structures (17.95 ± 0.65 mg GAE/g of extract and 16.53 ± 0.65 mg GAE/g of extract).

### 3.2. Biological activity

#### 3.2.1. Antioxidant Activity of *P. glauca*

One of the goals of our study was to assess the antioxidant potential of *P. glauca* extracts and caperatic acid, one of the main metabolites of the examined lichen species. To evaluate the various mechanisms of the tested substances’ antioxidant activity, we used differently in vitro methods. Therefore, the non-enzymatic antioxidant potential and the influence on the activity of antioxidant enzymes were evaluated.

##### ABTS and CUPRAC Analysis

For the investigation of the antiradical potential of *P. glauca* extracts, the ABTS technique was used. Most of the *P. glauca* extracts scavenged the ABTS^•+^, but the potent activity was significantly lower compared to the strong antioxidants such as vitamin C and quercetin (Table 2). The lowest activity was observed for the lipophilic DCM extract and non-aromatic caperatic acid (IC_50_ 0.381 mg/mL).

The next colorimetric antioxidant capacity assay performed using the *P. glauca* extracts was CUPRAC analysis. In this experiment, the capability of hydroxyl groups of polyphenolic compounds to be oxidized is mainly measured using the Cu^2+^-neocuproine complex [47]. Our results provide the highest DCM (IC_0.5_ 0.092 ± 0.001 mg/mL) and Ace (0.146 ± 0.002 mg/mL) extracts to reduce the Cu^2+^ cations. What is important is that the activity of the most active extract was only ~7–8 times less active than vitamin C. The activity between 0.245 mg/mL and 0.388 mg/mL was observed for the MeOH, H_2_O, or MeOH-H_2_O extracts. Caperatic acid showed a low Cu^2+^ reduction potential.

##### Cu^2+^ and Fe^2+^ Metal Chelating Ability

The excessive concentration of transition metal ions initiates the oxyradical generation and produces oxidative damage to the biomolecules [48]. In this manner, we tested the antioxidant effect of *P. glauca* extracts through transition metal ion chelation. We showed that *P. glauca* extracts are characterized by significant dose-dependent chelation potential, and quercetin was used as a reference substance. Independently of the examined metal ions, the strong activity, ~ 2.5–4 times weaker than reference, was proved for MeOH extract and MeOH-H_2_O extract. The DCM and Ace extracts, in dependence on the chelated ion, exhibited the highest activity of the tested extracts, and the potential was the same as the quercetin (for Cu^2+^: 14.7 ± 0.6 µg/mL, 12.0 ± 0.8 µg/mL, and 12.4 ± 0.4, respectively). The Fe^2+^ chelation potential of DCM extract and Ace extract was weak (for Fe^2+^: 1.83 ± 0.21 mg/mL, 4.0 mg/mL). Interestingly, *P. glauca* polar extracts were characterized by much stronger activity than quercetin (Table 3 and Table 4).

##### Impact on Reactive Oxygen Species (ROS) Homeostasis

The cellular defence against oxidative stress is related to the sufficient activity of antioxidant enzymes. Hence, the appropriate level of CAT, SOD, GPX, and GP allows you to reduce the probability of damaging cellular protein, carbohydrates and lipid peroxidation [49]. In this study, we examined the impact of *P. glauca* extracts and caperatic acid on the activity of antioxidant enzymes. The obtained results showed that all tested lichen substances inhibit the antioxidant enzymes in different modes. CAT was the highest inhabited by the H_2_O extract (70.8 ± 3.0%), which on the other hand, was the one that inhibited the weakly SOD (11.5 ± 1.9%). The different extracts influenced these two enzymes by ~30–40% (Table 5). The most potent enzyme inhibited was GPx, whose activity was decreased by 87.5 ± 3.2% when the H_2_O extract was used. Caperatic acid and MeOH-H_2_O were also detected as strong inhibitors of this enzyme. The weakly inhibited enzyme was GR (between 7.9 ±1.4 to 34.5 ±1.0%).

#### 3.2.2. Anticholinesterase Activity

##### AChE and BChE Inhibitory Effect

Our results proved that BChE was the more susceptible to lichen substance inhibition. High activity was noted for DCM extract, Ace extract (IC_50_ = 0.405 ± 0.009, 0.503 ± 0.057 mg/mL, respectively) and caperatic acid (IC_50_ = 0.610 ± 0.013 mg/mL), whereas the extracts with the higher polarity were characterised by lower inhibitory potential (Table 6). Among the six substances analysed in this study (extracts and caperatic acid), only the three with the highest inhibitory potential against BChE showed inhibitory activity on AChE. The most interesting activity against AChE was shown by caperatic acid (Table 6). Despite the detected inhibitory effect of *P. glauca* substances, these activities were weaker than galanthamine, used in the therapy of AD.

##### Molecular Docking on Cholinesterse

**Figure 3:**
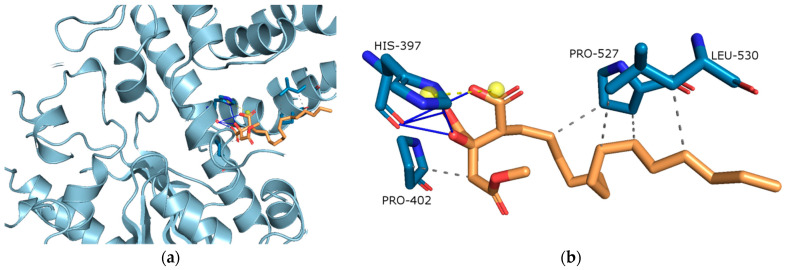
(**a**) Active site gorges of human acetylcholinesterase (AChE, PDB id: 4M0E); (**b**) Proposed binding mode of caperatic acid with AChE. The key interactions of caperatic acid with residues in the active sites of AChE. Hydrophobic interactions (grey dashed lines).

In order to get guidance for rational structural optimization of caperatic acid, the proposed binding mode of caperatic acid with the active sites of AChE and BChE was explored using a molecular docking method. After docking caperatic acid to the AChE (binding energy: −7.17 kcal/mol) revealed conspicuous (i) hydrophobic interactions between aliphatic chain and PRO-527, and LEU-530; and C atom in caperatic acid and PRO-402 (grey dashed lines); (ii) hydrogen bonds between carboxy and hydroxy group in caperatic acid and HIS-397 (blue lines), (iii) salt bridges (yellow line) (Figure 3b).

**Figure 4:**
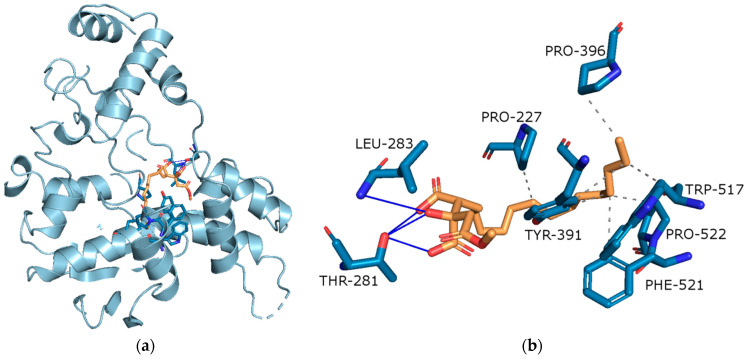
(**a**) Active site gorges of human butyrylcholinesterase (BChE, PDB id: 4BDS); (**b**) Proposed binding mode of caperatic acid with BChE. The key interactions of caperatic acid with residues in the active sites of BChE. Hydrophobic interactions and hydrogen bonds.

After docking caperatic acid to the BChE (binding energy: −9.08 kcal/mol) revealed conspicuous (i) hydrophobic interactions between aliphatic chain and PRO-227, PRO-396, PRO-522, TYR-391, TRP-517 and PHE-521 (grey dashed lines); (ii) hydrogen bonds between carboxy and hydroxy group in caperatic acid and LEU-283 and THR-281 (blue lines) (Figure 4b).

On the basis of binding energy, it can be indicated that BChe will be more strongly inhibited by caperatic acid.

#### 3.2.3. Anti-Inflammatory Activity

##### Inhibition of Cyclooxygenase-2 (COX-2)

Since COX-2 activity can is regarded as participating in the neuroinflammatory process [50], as well as this is overexpressed with a critical function in glioblastoma we decided to verify if the *P. glauca* extracts and its main secondary metabolite, caperatic acid, exert an impact on COX-2 activity. Our results proved that the *P. glauca* extracts could inhibit COX-2 between 4.8 ± 1.0% and 28.6 ± 0.3%. The COX-2 inhibitory effect of caperatic acid was one of the highest and was equal to 25.9 ± 0.1%. DCM extract was, as the alone, inactive tested substance (Table 7).

##### Anti-Hyaluronidase Activity

**Figure 5:**
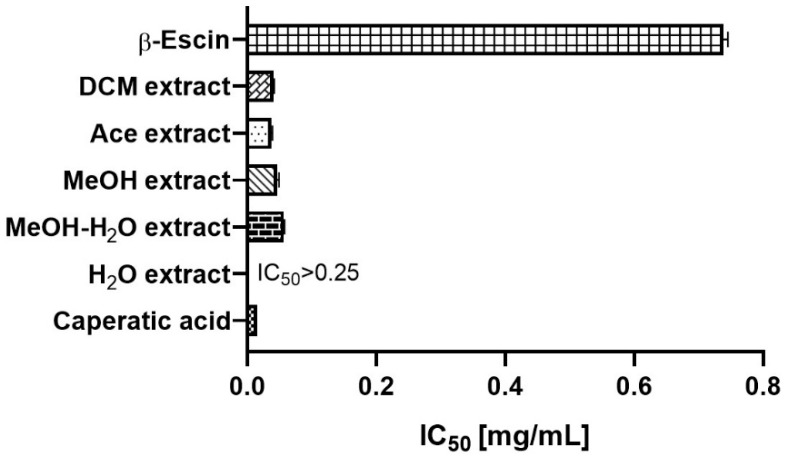
Inhibition of hyaluronidase by the *P. glauca* and extracts as well as by β-escin (served as a standard in this assay). Results are presented as IC_50_ mean values (mg/mL) ± SEM (*n* = 4—for *P. glauca* extracts, and caperatic acid and *n* = 6—for referenced: β-escin) obtained in two independent experiments.

Hyaluronidase is the hyaluronic acid degrading enzyme. Because a high-molecular weight-hyaluronan elicits anti-inflammatory and anti-proliferative responses, the activity of hyaluronidase may play an important role in the development of brain disorders [51]. Importantly, hyaluronidase is also highly expressed in patients with malignant glioma [52], and the high activity of hyaluronidase promotes the increase in the invasiveness of the malignant glioma cell [53]. Thus, to evaluate the inhibitory effect of *P. glauca* extract and caperatic acid on hyaluronidase activity, we used the in vitro method. Here, as shown in Figure 5, the high hyaluronidase inhibitory effect is demonstrated. Caperatic acid (IC_50_ = 0.016 ± 0.000 mg/mL) was more active than *P. glauca* extracts (the most active was DCM extract with IC_50_ = 0.041 ± 0.001 mg/mL). Importantly, β-escin, to reach the same level of enzyme inhibition, had to be tested in higher concentrations (IC_50_ = 0.738 ± 0.007 mg/mL). The results are obtained for the first time for the *P. glauca* extracts and caperatic acid.

#### 3.2.4. Anti-Tumour Activity

##### Cytotoxic Activity against GBM Cells

**Figure 6:**
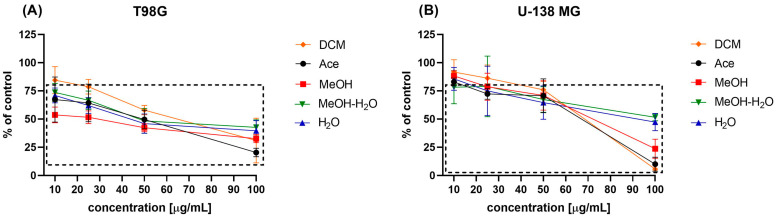
The effects of dichloromethane (DCM), acetone (Ace), methanol (MeOH), methanol-water (MeOH-H_2_O) and water (H_2_O) extracts of *P. glauca* on GBM cell viability (panel (**A**)—T89G cell line, panel (**B**)—U-138 MG cell line) after 48 h of treatment. Data presented as mean values ± SEM from three independent experiments. Statistically significant results different from the DMSO treated control are presented inside the rectangle.

The MTT test revealed that the tested extracts showed considerable cytotoxic effect on GBM cells. As presented in Figure 6, T98G cell line was more sensitive to the treatment with *P. glauca* extracts. All analysed extracts and their tested concentrations (except one case: 10 µg/mL DCM extract) significantly decreased cell viability. The most pronounced effect was observed for 100 µg/mL Ace extract, which reduced T98G cell viability to only 20.3 ± 3.79%, while the weakest effect (regarding the concentration range 10–50 µg/mL) was observed for DCM extract. The effect of 100 µg/mL DCM was similar to the one exerted by MeOH.

As far as U-138 MG cell line is concerned we also observed dose-dependent reduction in cells viability. The extracts in concentration of 10 µg/mL were insufficient to induce significant reduction in cells viability, but 25 µg/mL extracts were cytotoxic (the only exception was DCM extract). The extracts in the two higher concentrations tested, namely 50 µg/mL and 100 µg/mL all reduced significantly U-138 MG cells viability. In this case the most pronounced effects were observed after the treatment with DCM and Ace extracts.

The IC_50_ values, calculated based on the MTT results are presented in Table 8.

##### Pro-Apoptotic Activity

**Figure 7:**
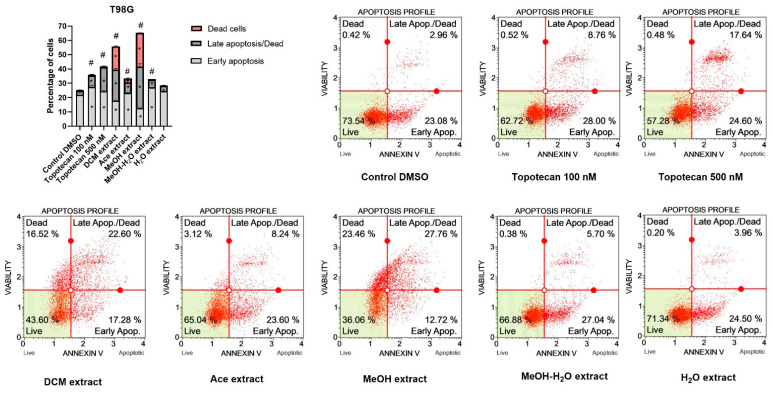
Apoptosis profiles of T98G cells as studied by the Muse™ Annexin V Dead Cell Kit after 48 h of treatment. Values on the bar chart are shown as mean ± SEM calculated from two independent experiments. (*) indicates statistically significant differences from control group for dead, late apoptosis/dead or early apoptosis cells; (#) above bar indicates statistically significant differences from control group for total apoptotic/dead cells, *p* 0.05. Representative histograms of controls (DMSO—negative control, topotecan—positive control) and analysed *P. glauca* extracts are also presented. Each histogram has four quadrant markers, reflecting the different cellular states: the upper left quadrant contains dead cells (necrosis), the upper right has late apoptotic/dead cells (cells that are positive both for Annexin V and for 7-Aminoactinomycin D), the lower left contains live cells and the lower right early apoptotic cells (cells that are positive only for Annexin V).

The results of flow cytometry analysis (Figure 7) revealed that *P. glauca* extracts induce apoptotic cell death in GBM cells. In T98G cell line, the strongest pro-apoptotic effect was obtained after the treatment with MeOH and DCM extracts. In both cases, the percentage of apoptotic cells reached ~40%. Moreover the treatment with these two extracts resulted in large amount of dead cells. Significant increase in both early and late apoptotic cells was also observed after the treatment with Ace and MeOH-H_2_O extracts. On the other hand, H_2_O extract increased only late apoptotic/dead cells fraction.

**Figure 8:**
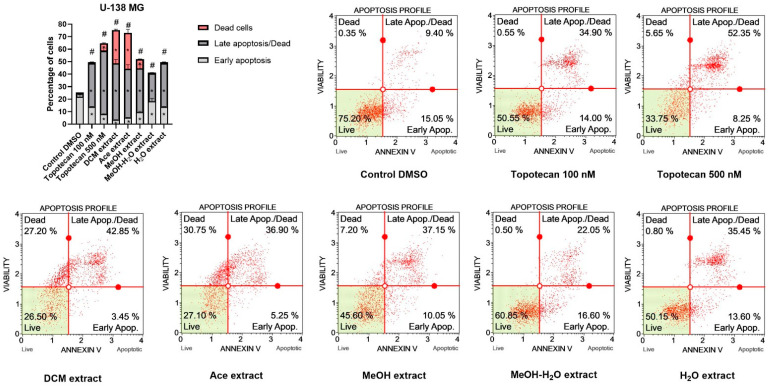
Apoptosis profiles of U-138 MG cells as studied by the Muse™ Annexin V Dead Cell Kit after 48 h of treatment. Values on the bar chart are shown as mean ± SEM calculated from two independent experiments. (*) indicates statistically significant differences from control group for dead, late apoptosis/dead or early apoptosis cells; (#) above bar indicates statistically significant differences from control group for total apoptotic/dead cells, *p* 0.05. Representative histograms of controls (DMSO—negative control, topotecan—positive control) and analysed *P. glauca* ex-tracts are also presented. Each histogram has four quadrant markers, reflecting the different cellular states: the upper left quadrant contains dead cells (necrosis), the upper right has late apoptotic/dead cells (cells that are positive both for Annexin V and for 7-aminoactinomycin D), the low-er left contains live cells and the lower right early apoptotic cells (cells that are positive only for Annexin V).

Generally, similar effects were obtained for U-138 MG cell line (Figure 8). In this case all extracts of *P. glauca* induced apoptosis, and the effect was comparable or stronger than 100 nM or even 500 nM topotecan, used as a positive control. In this cell line we observed the most pronounced apoptotic cell death of GBM cells after the treatment with DCM, Ace and H_2_O extracts; however, the first two mentioned extracts also increased the percentage of necrotic cells, whereas H_2_O extract did not cause such effect. We observed dead cells also after the treatment with MeOH extract and 500 nM topotecan. Interestingly, the treatment with MeOH-H_2_O extract of *P. glauca* resulted in the highest percentage of early apoptotic cells.

##### Impact on Cell Cycle Distribution of GBM Cells

**Figure 9:**
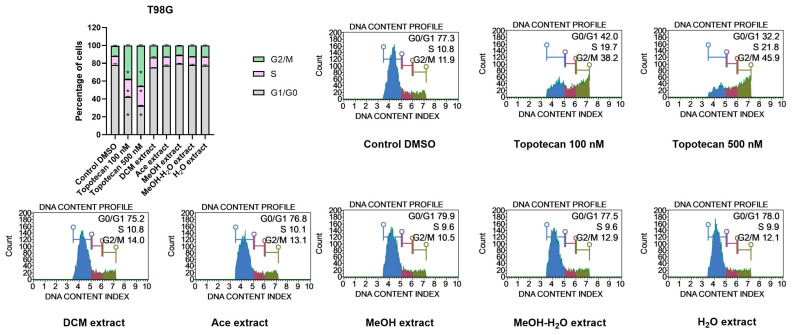
Cell cycle analysis of T98G cells using the Muse™ Cell Cycle Kit after 48 h of treatment. The percentage of cells in G0/G1, S, and G2/M phases of the cell cycle are presented in the bar chart. The values are shown as the mean ± SEM calculated from two independent experiments. (*) indicates statistically significant differences from the control group for a particular phase, *p* 0.05. Representative histograms of the controls (DMSO—negative control, topotecan—positive control) and analysed *P. glauca* extracts are also presented.

**Figure 10:**
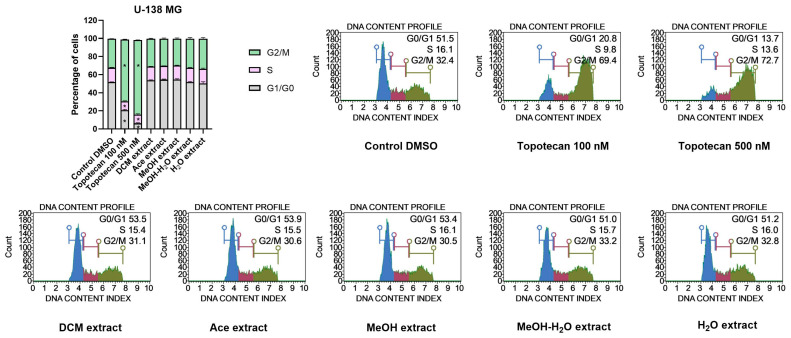
Cell cycle analysis of U-138 MG cells using the Muse™ Cell Cycle Kit after 48 h of treatment. The percentage of cells in G0/G1, S, and G2/M phases of the cell cycle are presented in the bar chart. The values are shown as the mean ± SEM calculated from two independent experiments. (*) indicates statistically significant differences from the control group for a particular phase, *p* 0.05. Representative histograms of the controls (DMSO—negative control, topotecan—positive control) and analysed *P. glauca* extracts are also presented.

Topotecan, used as a positive control in the cell cycle assay increased the number of cells in the G2/M phases. Higher concentration of the tested topotecan (500 nM) created stronger shift towards G2/M arrest, as compared to 100 nM topotecan. However, despite the strong pro-apoptotic effect induced by *P. glauca* we did not observe any changes in cell cycle phases distribution after the treatment with 50 µG/mL extracts. Neither in T98G, nor in U-138 MG cells the extracts modified the distribution of G0/G1, S and G2/M phases (Figure 9 and Figure 10).

#### 3.2.5. Summary of Biological Potential of Lichen-Derived Compounds and Extracts

**Figure 11:**
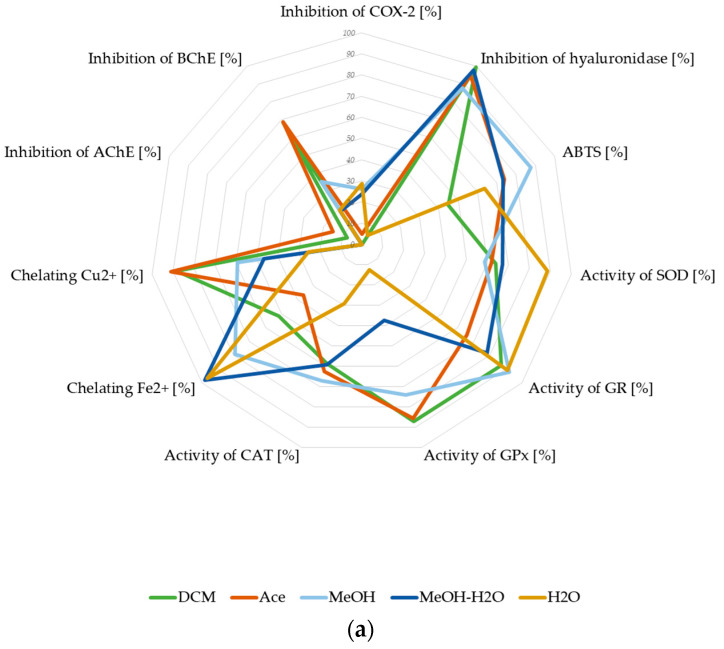

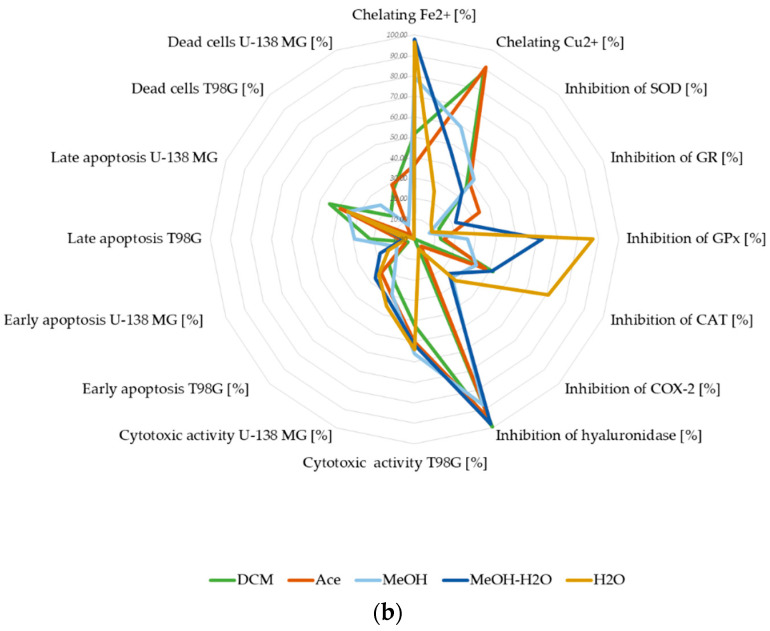
The biological potential of *P. glauca* extracts: neuroprotective potential (**a**) and anti-GBM potential (**b**), expressed in % of appropriate types of biological activity. The graphs were made for the final concentrations: ABTS^•+^ 0.381 mg/mL (**a**); inhibition of AChE 0.800 mg/mL (**a**,**b**); inhibition of BChE 0.800 mg/mL (**a**); chelating Fe^2+^ 1.6 mg/mL (**a** and **b**); chelating Cu^2+^ 0.4 mg/mL; inhibition of COX-2 0.348 mg/mL (**a**,**b**); inhibition of hyaluronidase 1.25 mg/mL (**a**,**b**); activity/inhibition of SOD 0.250 mg/mL (**a**,**b**); activity/inhibition of GR 0.124 mg/mL (**a**,**b**); activity/inhibition of GPx 0.328 mg/mL (**a**,**b**); activity/inhibition of CAT 0.102 mg/mL (**a**,**b**); cytotoxicity expressed as % of cell death: U-138 mg 0.05 mg/mL (**b**), and T98G 0.05 mg/mL (**b**); early apoptosis U-138 MG 0.05 mg/mL; early apoptosis T98G 0.05 mg/mL. The activity of antioxidants enzymes was calculated from the difference between 100% of enzyme activity and % of the experimentally evaluated enzyme inhibition.

To summarize our study, we put the biological activity results of *P. glauca* extracts on the star diagram. The presented charts allow us to compare the neuroprotective potential of extracts as well as the anti-GBM properties. The analysis of the first graph (Figure 11a) proved that neuroprotective potential could be mainly connected with the antioxidant activity measured by different mechanisms (ABTS, chelating of metal ions activity, and the low percent of inhibition of antioxidant activity enzymes). The ability to inhibit AChE and BChE was lower and in favour of the BChE. The anti-inflammatory action was strongly represented by the anti-hyaluronidase activity. Moreover, as presented in Figure 11b, the cytotoxicity and pro-apoptotic activity contribute to the anti-GBM properties of *P. glauca* extracts. Although both tested GBM cell lines responded in a similar manner to the treatments, the chart shows that the anti-tumour activity of *P. glauca* extracts was more pronounced in T98G cells than in U-138 MG. Furthermore, the diagram also shows that the chelating and anti-hyaluronidase activity could also play essential roles in the antineoplastic activity of examined lichen extracts. Regarding the star diagram, we assumed that the smaller contribution in the anti-GBM potential was for COX-2 and inhibition of antioxidant enzymes (especially SOD and GR).

The mathematical addition of the % of biological activity, considering the number of variables in each assessed activity (Table 9), indicates the general trend of bioactivity of the examined *P. glauca* extracts. Overall, all tested extracts exhibited some neuroprotective properties, and MeOH, Ace and DCM extracts were the most active in this context. Regarding the anti-GBM potential, the MeOH-H_2_O, MeOH extracts showed the most promising properties; however, DCM and Ace extracts are also interesting. Therefore, they should be investigated in further studies.

## 4. Discussion

The number of literature reports on the evaluation of biologic potential of *P. glauca* is limited. Thus, our study aimed to investigate the biological activity of *P. glauca* extracts as well as the activity of *P. glauca* major compound caperatic acid—in order to evaluate their potential to support the treatment of the CNS diseases through the neuroprotective and anti-cancer effects.

Phytochemical analysis of *P. glauca* extracts was performed using infrared spectroscopy (FT-IR), and gas chromatography-mass spectrometry analysis (GC-MS). Moreover, total polyphenol content was determined spectroscopically. The FT-IR study proved that *P. glauca* extracts contain atranorin, methyl β-orcinolcarboxylate and caperatic acid. Owing to the second derivative spectroscopy application, FT-IR analysis was used as a tool to confirm the presence of the selected compound in the studied extract. A similar analysis has already been applied to study the plant extracts [44]. The chemical profile of the volatile components in the *P. glauca* extracts was also determined by GC-MS chromatographic analysis. Moreover, we found that MeOH extract has the highest polyphenols content (39.11 ± 1.19 mg GAE/g of extract). This value is almost identical to that of the methanol extract reported by Mitrović et al. (2014) (39.75 mg GAE/g) [16]. In another study, polyphenols content in *P. glauca* methanol extract was 1.1% [20], which is lower as compared to our study (3.91%). Moreover, in the same study, the acetone extract of *P. glauca* contained higher, as compared to our results, polyphenol content (63.69 mg GAE/g). These discrepancies may be caused by the different environmental factors influencing the production of secondary metabolites in the lichen. This effect has already been described in the literature [54,55].

Effective protection against oxidative stress is one of the critical factors for proper CNS function. Because of the high metabolic rate in neurons, the CNS produces significant amounts of the reactive oxygen species (ROS) [56]. It has been proven that oxidative imbalance induces neuron degeneration, which is closely associated with neuronal aging and neuronal disorders [56]. Lichens have developed the ability to reduce the antioxidant stress generated by ROS [57]. Our results confirm that *P. glauca* extracts are characterized by different antioxidant potentials. Using the DPPH assay, Mitrović et al. (2014) indicated that the methanol extract of *P. glauca* had a higher ROS scavenging activity than the more lipophilic extracts [16]. In our study, the scavenging activity was measured using the ABTS^+•^ method. We showed that the antioxidant activity of MeOH extract was the highest, as compared to other evaluated extracts. The content of polyphenols in the extract corresponds with its antioxidant activity [58]. *P. glauca* MeOH extract was the richest in polyphenolic compounds among the ones examined. According to our results, all tested *P. glauca* extracts exert some antioxidant activity; however, caperatic acid did not show the antioxidative effects. According to our best knowledge, this is the first report showing no antioxidant activity of this substance.

The deficiency or the excess of metal ions is harmful to the homeostasis of cells [59]. It is related to the redox reactions carried out by metal cations (mainly Fe^2+^ and Cu^2+^) which generate ROS. Failure to control the redox homeostasis can result in biomolecular damage, leading to cell death. It has been shown that Cu^2+^ and Fe^2+^ metal dyshomeostasis may correlate with serious diseases, including those with a neurodegenerative background. Moreover, it has also been demonstrated that β-amyloid condensation, characteristic for AD is associated with high Cu^2+^ levels. Moreover, it was reported that binding of Cu^2+^ to the α-synuclein protein is important in the progression of Parkinson’s disease. The increased level of Cu^2+^ can also be related to cancer initiation or progression [59,60,61]. Many substances of natural origin can selectively bind metal ions, serving as free radical scavengers [60,61,62]. We have shown that *P. glauca* extracts have a significant chelating activity, and the chelating potential of individual extracts differs depending on the metal tested. Literature indicates that lichens can serve as metal chelators [63] due to the production of metabolites playing a role in metal complexation [64]. This capability depends on the lichen metabolites and cation type [63,64] and ensures the survival of lichens in harsh environmental conditions [65]. For example, depsidone produced by *Hypogymnia physdes*—physodalic acid, can uptake Cu^2+^ but not Fe^2+^ [66,67], whereas Cu^2+^ uptake is also promoted by other lichenic compounds, (+)-usnic acid or divaricatic acid [68]. According to our best knowledge, the ability to chelate metal ions by *P. glauca* extracts has not been tested so far. It was only found that atranorin, depside present in *P. glauca*, can capture Cu^2+^ ions (37%), while the uptake of Fe^2+^ ions by atranorin was not observed [66]. The earlier published data are consistent with our results. Here we show that the lipophilic extracts of *P. glauca* contain compounds from the group of depsides (e.g., atranorin) that can capture Cu^2+^ ions. On the other hand, polar extracts proved to chelate Fe^2+^ ions more powerfully, which suggests their different chemical composition. This activity confirms the neuroprotective potential of *P. glauca* extracts [69].

SOD, CAT, GR and GPx are the cells antioxidant enzymes [70]. Their low level in brain tissue may result in the oxidative damage caused by ROS [71] and induce neurodegenerative disorders [72]. Moreover, it was found that these enzymes are expressed in brain tumours [73]. In addition, recent data indicate that a low level of intracellular antioxidant enzymes in cancer cells [70] improves the effectiveness of brain tumours treatment [15,74]. In this work, we show, for the first time, that *P. glauca* extracts and caperatic acid can inhibit the activity of intracellular antioxidant enzymes to varying degrees. Thus, *P.glauca* extracts should be further analysed as a potential adjuvant to standard anti-GBM therapy. Our earlier works also indicated that the other lichen extracts and compounds are characterized by antioxidant enzyme inhibitory potential. We showed that acetone extract from *Evernia prunastri* strongly inhibited SOD (53.4 ± 2.4%), GR (91.1 ± 7.2%) and GPx (92.4 ± 4.3%) [13]. Moreover, the ability to inhibit SOD and GPx for acetone extracts of *P. sulcata* and *C. uncialis* was demonstrated. The lichen depside (evernic acid), depsidone (salazinic acid), and dibenzofuran ((−)—usnic acid) inhibited SOD and selectively GR or GPx [13].

Patients with AD have reduced levels of acetylcholine in the brain [75]. Cholinesterases (ChEs) regulate the correct level of acetylcholine in the CNS, while modulation of ChEs activity influences therapy response. AChE and BChE were found in neurons, glial and neuritic plaques, or tangles of AD patients. Moreover, AD progression is accompanied by a decrease in AChE activity, which is compensated by the increase in the BChE activity. ChEs also influence the synthesis, deposition and aggregation of toxic β-amyloid. Little is known about the role of ChEs in GBM. Some papers show significant enhancement of ChE activity in the tumour cells and brain glioma [76]. On the other hand, acetylcholine has been identified as a modulator of GBM behaviour in a way that GBM cells may utilise acetylcholine as an autocrine signalling molecule [77]. Thus, the importance of ChEs inhibitors in developing GBM is unclear. Numerous reports identify plant cholinesterase inhibitors [78,79]. However, little is known about the anticholinesterase activity of lichen compounds and extracts. The results of our study indicate that *P. glauca* and caperatic acid have the anticholinesterase activity, especially in relation to BChE. A similar tendency was observed in our previous study, in regard to BChE, not AChE, which were mildly inhibited by physodic acid, evernic acid and salazinic acid [13,14]. Moreover, in other study, AChE was inhibited by perlatolic acid [80]. The acetone extracts from lichens *Evernia prunastri*, *Parmelia sulcata* and *Cladonia uncialis* inhibited BChE only. Furthermore, *Hypogymnia physodes* and *Cladonia uncialis* acetone extract inhibited AChE [13,14]. Our in silico studies are consistent with in vitro experiments and confirm the ability of caperatic acid to inhibit the activity of AChE and BChE enzymes. It was the first report on the anticholinesterase activity of caperatic acid. In our study we provide evidence that *P. glauca* extracts have anticholinesterase activity, and therefore should be further investigated in the context of AD.

The increased activity of COX-2 and hyaluronidase, are associated with the inflammatory process accompanying diseases of various aetiologies [81,82] including neurodegenerative diseases and CNS neoplasms [81,83]. Literature data indicate that COX-2 is overexpressed in GBM, and hyperactive hyaluronidase can increase GBM cell invasion [84,85]. Hence, in this study we verified whether *P. glauca* extracts and caperatic acid have anti-COX-2 and anti-hyaluronidase activity. The results of our study proved for the first time that caperatic acid inhibits both COX-2 and hyaluronidase activity. Moreover, COX-2 and hyaluronidase were also inhibited by most of the tested extracts. The ability of lichens to inhibit COX-2 and hyaluronidase has already been described in the literature by other authors and us. In our previous study, we found that COX-2 was inhibited by salazinic acid, evernic acid and (−)-usnic acid. The same compounds also inhibited hyaluronidase (IC_50_ from ~0.5 to ~0.6 mg/mL). However, it has to be noted that the results of caperatic acid and *P. glauca* extracts indicated their stronger COX-2 and anti-hialuronidase activity, as compared to salazinic acid, evernic acid and (-)-usnic acid [13].

Almost 20 year ago the cytotoxic activity of some lichen extracts on human cancer cell lines was reported [22]. More recently we also demonstrated cytotoxic and pro-apoptotic potential of the lichen secondary metabolites and lichen-derived extracts against GBM cells [13,14,15]. However, data about the anticancer potential of *P. glauca* extracts are scarce. Here we showed a considerable cytotoxic effect of *P. glauca* extracts on GBM cells. After 48 h, the highest cytotoxicity was exerted by MeOH extract (IC_50_ = 31.8 ± 10.4 µg/mL) on T98G cell line, and Ace extract (IC_50_ = 65.7 ± 5.0 µg/mL) on U-138 MG cells, respectively. We also observed the strong pro-apoptotic/necrotic activity of the tested extracts. In this regard, the highest number of apoptotic/dead cells was reported for MeOH in T98G cell line, and DCM and Ace extracts in U-138 MG cell lines, which is in line with their huge cytotoxic potential. Similar results were published by Šeklić et al. (2018) [21], who found that *P. glauca* extracts induced cytotoxic effects on HCT-116 colorectal cancer cell line after 72 h (IC_50_ 40 μg/mL), while methanol and acetone extracts had cytotoxic effects on SW-480 cells after 24 h. They also observed the pro-apoptotic/necrotic activity of the tested extracts, which was a consequence of induced oxidative stress. Moreover, anti-migratory and anti-invasive properties of *P. glauca* were also reported in more recent studies [17,86]. As COX-2/PGE2/EP4 pathway is crucial for GBM cells, strong inhibition of PGE2 formation exerted by *P. glauca* extracts might also be the factor determining strong pro-apoptotic potential observed in our study [17,85]. However, we did not see any impact of the studied extracts in regard to cell cycle distribution. This is in line with the study of Ingelfinger et al., who also did not report any changes in the distribution of the cell cycle phases in a model of colorectal cancer cells [17].

## 5. Conclusions

Our study shows that *P. glauca* extracts, besides caperatic acid, contain other compounds, including atranorin and methyl β-orcinolcarboxylate. The results of our analysis provide evidence that *P. glauca* extracts have antioxidant, AChE and BChE inhibitory, as well as anti-inflammatory properties. The five *P. glauca* extracts analysed exert similar neuroprotective potential. However, among the five *P. glauca* extracts, the MeOH, Ace and DCM extracts should be further analysed, as according to our study they are the most suitable for the adjuvant treatment of neurodegenerative diseases. Furthermore, we also provided evidence for the cytotoxic and pro-apoptotic effects of *P. glauca* extracts, and we found them as the active substances. Thus, they should be further analysed as an adjuvant to the standard anti-GBM treatment. In conclusion, our study shows that *P. glauca* is a lichen with valuable neuroprotective and anti-cancer effects and may potentially offer new adjuvant treatment options for patients with AD and GBM.

## Figures and Tables

**Table 1:** Compounds identified in the extracts of *P. glauca* using GC-MS.

Extract	Rt (min.)	Compounds	% of Total	MW	Formula
DCM extract	6.305	2,2,6-trimethyloctane	3.63	156.31	C_11_H_24_
	6.559	2,2,3,5-tetramethylheptane	0.85	156.31	C_11_H_24_
	6.886	2,3,3-trimethyloctane	0.62	156.31	C_11_H_24_
	6.928	3,3,5-trimethylheptane	0.49	142.28	C_10_H_22_
	7.043	benzeneacetic acid, 3-tetradecyl ester	0.24	332.50	C_22_H_36_O_2_
	7.431	2,2,4,6,6-pentamethylheptane	6.87	170.33	C_12_H_26_
	7.659	2,3,6,7-tetramethyloctane	4.55	170.33	C_12_H_26_
	7.706	2,5,9-trimethyldecane	1.07	184.37	C_13_H_28_
	7.798	5,6-dimethyldecane	0.21	170.33	C_12_H_26_
	7.997	5-ethyl-2,2,3-trimethylheptane	7.11	170.33	C_12_H_26_
	8.034	2,6,7-trimethyldecane	0.73	184.37	C_13_H_28_
	8.324	5-nonanone	60.01	142.24	C_9_H_18_O
	8.377	decane	2.72	142.28	C_10_H_22_
	8.417	4-ethyl-2,2-dimethylhexane	2.19	142.28	C_10_H_22_
	8.545	3,7-dimethyldecane	0.97	170.33	C_12_H_26_
	8.626	2,2,4-trimethyl-5-hexen-3-ol	0.71	142.24	C_9_H_18_O
	8.691	2,4-dimethylundecane	0.47	184.37	C_13_H_28_
	21.688	1,2,3,5,6,7-hexahydro-1,1,4,8-tetramethyl-s-indacene	5.59	214.35	C_16_H_22_
	25.641	9,9a-bis(acetyloxy)-1,1a,1b,4,4a,7a,7b,8,9,9a-decahydro-4a,7b-dihydroxy-1,1,6,8-tetramethyl-3-[(triphenylmethoxy)methyl]-, [1aR-(1aa,1bß,4aß,7aa,7ba,8a,9ß,9aa)]-5*H*-cyclopropa[3,4]benz[1,2-e]azulen-5-one	0.97	690.83	C_43_H_46_O_8_
Total			100.00		
Ace extract	3.231	triethyl borate	12.24	145.99	C_6_H_15_BO_3_
	17.205	benzoic acid, 2,4-dihydroxy-3,6-dimethylmethyl ester methyl ester	46.50	196.20	C_10_H_12_O_4_
	17.504	atranorin	4.34	374.35 374.35 374.35	C_19_H_18_O_8_
	18.952	2-heptadecanone	1.63	254.45	C_17_H_34_O
	19.174	hexadecanoic acid, methyl ester	0.54	270.45	C_17_H_34_O_2_
	22.757	*E*,*E*,*Z*-1,3,12-nonadecatriene-5,14-diol	3.43	294.47	C_19_H_34_O_2_
	23.057	12-methyl-E,E-2,13-octadecadien-1-ol	2.75	280.49	C_19_H_36_O
	24.900	spiculesporic acid	27.17	328.40	C_17_H_28_O_6_
	25.039	ethyl iso-allocholate	1.40	436.62	C_26_H_44_O_5_
Total			100.00		
MeOH extract	8.340	acetophenone	23.25	120.15	C_8_H_8_O
	13.419	(*R*)-α-methylbenzenemethanol	0.36	122.16	C_8_H_10_O
	14.854	2,3,3-trimethyl-5-phenyl-1-pentene	17.52	188.31	C_14_H_20_
	18.797	3-phenylbutyrophenone	22.46	224.30	C_16_H_16_O
	20.132	1,3-diphenyl-2-buten-1-one	36.41	222.28	C_16_H_14_O
Total			100.00		
MeOH-H_2_O extract	14.859	2,3,3-trimethyl-5-phenyl-1-pentene	41.56	188.31	C_14_H_20_
	14.923	(R*,R*)-5-nitro-1-phenyl-1-hexen-3-ol	2.04	221.25	C_12_H_15_NO_3_
	18.842	3-phenylbutyrophenone	43.36	224.30	C_16_H_16_O
	20.219	1,3-diphenyl-2-buten-1-one	9.65	222.28	C_16_H_14_O
	25.617	1,4-diol-1,4-diphenyl-2-butene	3.39	240.30	C_16_H_16_O_2_
Total			100.00		
H_2_O extract	7.703	2-(2-(2-methoxyethoxy)ethoxy)ethyl 2-methylbutanoate	5.66	248.32	C_12_H_24_O_5_
	9.653	4,5-dihydro-5-thioxo-1,2,4-triazin-3(2H)-one	17.77	129.14	C_3_H_3_N_3_OS
	12.722	4,4,5,5-tetramethyl-2-phenyl-1,3,2-dioxaborolane	14.28	204.07	C_12_H_17_BO_2_
	13.531	4-octadecenal	2.90	266.46	C_18_H_34_O
	14.003	9-oximino-2,7-diethoxyfluorene	2.59	283.33	C_17_H_17_NO_3_
	21.523	triphenylphosphine	56.81	262.29	C_18_H_15_P
Total			100.00		

**Table 2:** Antioxidant activity of lichen extracts, caperatic acid and quercetin and vitamin C as the references measured using ABTS and CUPRAC methods.

Extract/Substance	ABTS Antiradical Activity [%]						CUPRAC Activity
	Concentration [mg/mL]					IC_50_ [mg/mL]	IC_0.5_ [mg/mL]
	0.024	0.048	0.095	0.190	0.381		
DCM extract	25.88 ± 0.39 ^c^	35.87 ± 0.36 ^b^	41.71 ± 0.40 ^b^	42.56 ± 0.33 ^c^	44.61 ± 0.71 ^d^	0.381 ^f^	0.092 ± 0.00 ^a^
Ace extract	27.01 ± 0.44 ^a^	38.18 ± 0.26 ^a^	44.52 ± 0.18 ^a^	51.92 ± 0.22 ^b^	73.92 ± 0.28 ^b^	0.155 ± 0.004 ^c^	0.146 ± 0.002 ^c^
MeOH extract	19.32 ± 0.32 ^b^	28.79 ± 0.28 ^c^	41.78 ± 0.42 ^b^	63.23 ± 0.59 ^a^	87.64 ± 0.19 ^a^	0.129 ± 0.004 ^b^	0.245 ± 0.001 ^d^
MeOH-H_2_O extract	10.36 ± 0.27 ^c^	18.16 ± 0.74 ^d^	32.03 ± 0.50 ^c^	51.99 ± 1.27 ^b^	73.49 ± 0.65 ^b^	0.178 ± 0.013 ^d^	0.388 ± 0.002 ^f^
H_2_O extract	7.67 ± 0.19 ^d^	13.93 ± 0.24 ^e^	22.85 ± 0.49 ^d^	39.73 ± 0.31 ^d^	63.75 ± 0.82 ^c^	0.260 ± 0.007 ^e^	0.325 ± 0.001 ^e^
Caperatic acid	2.81 ± 0.93 ^e^	4.56 ± 0.35 ^f^	8.24 ± 0.14 ^e^	17.84 ±0.36 ^e^	31.67 ± 0.62 ^e^	0.381 ^f^	0.882 ± 0.051 ^g^
Quercetin	nt					0.002 ± 0.000 ^a^	nt
Vitamin C	nt					0.006 ± 0.000 ^a^	0.012 ± 0.000 ^a^

Mean values within a column with the same letter are not significantly different at *p* 0.05 using Duncan’s test. The first letter of the alphabet for the highest values, the next for statistically significant decreasing values. The mean values ± SEM from *n* = 6 independent measurements; nt—not tested at this concentration.

**Table 3:** Cu ^2+^ chelating activity of lichen extracts and quercetin as the reference.

Extracts/ Substance	Chelating Cu^2+^ [%]						IC_50_ [µg/mL]
	Concentration [mg/mL]						
	0.005	0.01	0.02	0.04	0.08	0.16	
DCM extract	18.7 ± 0.2 ^b^	35.1 ± 1.2 ^b^	58.8 ± 2.0 ^b^	88.4 ± 2.6 ^b^	96.0 ± 0.8 ^a^	94.7 ± 1.2 ^ab^	14.7 ± 0.6 ^a^
Ace extract	26.3 ± 1.5 ^a^	43.7 ± 0.6 ^a^	71.4 ± 0.3 ^a^	91.2 ± 1.3 ^ab^	96.9 ± 0.5 ^a^	96.1 ± 0.3 ^ab^	12.0 ± 0.8 ^a^
MeOH extract	nt	25.3 ± 1.4 ^c^	38.7 ± 4.6 ^c^	59.2 ± 4.4 ^c^	94.7 ± 0.5 ^a^	96.7 ± 0.1 ^ab^	29.8 ± 2.4 ^ba^
MeOH-H_2_O extract	nt	17.3 ± 0.1 ^d^	28.2 ± 1.3 ^d^	46.7 ± 1.6 ^d^	72.3 ± 3.6 ^b^	90.3 ± 0.7 ^b^	45.7 ± 3.1 ^b^
H_2_O extracta	nt	10.5 ± 0.2 ^e^	16.8 ± 1.8 ^e^	25.4 ± 3.1 ^e^	43.6 ± 0.9 ^c^	51.1 ± 1.6 ^c^	141.0 ± 17.4 ^c^
Quercetin	26.0 ± 2.0 ^a^	44.3 ± 0.5 ^a^	72.7 ± 0.9 ^a^	97.5 ± 1.0 ^a^	97.7 ± 0.1 ^a^	98.5 ± 5.2 ^a^	12.4 ± 0.4 ^a^

Mean values within a column with the same letter are not significantly different at *p* 0.05 using Duncan’s test. The first letter of the alphabet for the highest values, the next for statistically significant decreasing values. The mean values ± SEM from *n* = 4 independent measurements; nt—not tested at this concentration.

**Table 4:** Fe ^2+^ chelating activity of lichen extracts and quercetin as the reference.

Extracts/ Substance	Chelating Fe^2+^ [%]					IC_50_ [mg/mL]
	Concentration [mg/mL]					
	0.08	0.16	0.8	1.6	4.0	
DCM extract	7.9 ± 0.3 ^c^	13.8 ± 0.7 ^c^	35.6 ± 0.8 ^d^	51.7 ± 3.6 ^c^	61.4 ± 1.3 ^c^	1.83 ± 0.21 ^d^
Ace extract	6.5 ± 0.1 ^dc^	7.0 ± 1.3 ^d^	20.7 ± 0.2 ^e^	36.6 ± 5.6 ^d^	40.0 ± 3.2 ^d^	4.0 ^e^
MeOH extract	10.9 ± 0.5 ^b^	16.9 ± 1.5 ^c^	52.9 ± 2.7 ^b^	79.3 ± 2.2 ^b^	96.1 ± 0.3 ^a^	0.55 ± 0.02 ^b^
MeOH-H_2_O extract	36.0 ± 1.3 ^a^	64.2 ± 1.8 ^a^	90.9 ± 2.4 ^a^	98.0 ± 0.2 ^a^	100.2 ± 0.9 ^a^	0.10 ± 0.01 ^a^
H_2_O extract	34.4 ± 0.9 ^a^	48.2 ± 2.5 ^b^	92.8 ± 0.5 ^a^	96.5 ± 0.3 ^d^	99.2 ± 0.5 ^a^	0.15 ± 0.01 ^a^
Quercetin	5.2 ± 0.9 ^d^	16.2 ± 0.8 ^c^	42.5 ± 1.0 ^c^	54.2 ± 1.7 ^c^	72.6 ± 1.8 ^b^	1.17 ± 0.03 ^c^

Mean values within a column with the same letter are not significantly different at *p* 0.05 using Duncan’s test. The first letter of the alphabet for the highest values, the next for statistically significant decreasing values. The mean values ± SEM from *n* = 4 independent measurements (extracts) or from *n* = 6 independent measurements (quercetin).

**Table 5:** Effect of lichen extracts and compounds on antioxidant enzymes.

Extract/Substance	CAT Inhibition Under Reaction Conditions (%)	SOD Inhibition Under Reaction Conditions (%)	GR Inhibition under Reaction Conditions (%)	GPx Inhibition under Reaction Conditions (%)
DCM extract	41.5 ± 3.5 ^b^	36.1 ± 2.2 ^b^	13.0 ± 2.1 ^c^	12.8 ± 2.4 ^e^
Ace extract	37.5 ± 4.4 ^bc^	38.3 ± 2.5 ^ab^	34.5 ±1.0 ^a^	14.5 ± 3.9 ^e^
MeOH extract	32.8 ± 3.1 ^bc^	41.3 ± 3.0 ^a^	7.9 ±1.4 ^d^	26.0 ± 4.1 ^d^
MeOH-H_2_O extract	40.9 ± 1.6 ^b^	33.0 ± 1.1 ^c^	21.7 ± 0.9 ^b^	62.5 ± 2.0 ^c^
H_2_O extract	70.8 ± 3.0 ^a^	11.5 ± 1.9 ^e^	9.2 ± 2.1 ^d^	87.5 ± 3.2 ^a^
Caperatic acid	30.7 ± 5.3 ^bc^	24.2 ± 0.8 ^d^	19.4 ± 2.3 ^b^	79.5 ± 3.8 ^b^

Mean values within a column with the same letter are not significantly different at *p* 0.05 using Tukey’s HSD test. The first letter of the alphabet for the highest values, the next for statistically significant decreasing values. The mean values ± SD from *n* = 4 independent measurements (extracts) or from *n* = 6 independent measurements (quercetin).

**Table 6:** Inhibition of AChE and BChE by lichen extracts and caperatic acid.

Extract/ Substances	AChE Inhibition [%]			BChE Inhibition [%]		
	Concentration [mg/mL]		IC_50_ [mg/mL]	Concentration [mg/mL]		IC_50_ [mg/mL]
	0.4	0.8		0.4	0.8	
DCM extract	na	7.65 ± 1.35 ^c^	nc	50.57 ± 0.81 ^a^	68.56 ± 1.45 ^a^	0.405 ± 0.009 ^b^
Ace extract	na	14.82 ± 0.96 ^b^	nc	37.69 ± 0.68 ^b^	68.58 ± 0.22 ^a^	0.503 ± 0.057 ^c^
MeOH extract	na	na	nc	21.70 ± 1.22 ^d^	35.21 ± 1.27 ^b^	0.800 ^e^
MeOH-H_2_O extract	na	na	nc	6.64 ± 1.50 ^f^	18.64 ± 1.32 ^c^	0.800 ^e^
H_2_O extract	na	na	nc	11.60 ± 1.60 ^e^	19.13 ± 0.91 ^c^	0.800 ^e^
Caperatic acid	3.04 ± 0.72	30.98 ± 2.29 ^a^	nc	31.11 ± 0.88 ^c^	69.11 ± 1.93 ^a^	0.610 ± 0.013 ^d^
Galantamine	nt		0.00046 ± 0.00004	nt		0.004 ± 0.000 ^a^

Mean values within a column with the same letter are not significantly different at *p* 0.05 using Duncan’s test. The first letter of the alphabet for the highest values, the next for statistically significant decreasing values. The mean values ± SEM from *n* = 6 independent measurements; na—not active; nt—not tested at this concentration; nc—not calculated at examined concentrations.

**Table 7:** Inhibition of cyclooxygenase-2 (COX-2) enzyme by the extracts of *P. glauca* and caperatic acid.

Extract/Substance	Equivalent Concentration of Acetylsalicylic Acid [mg/mL]	COX-2 Inhibition under Reaction Conditions [%]
DCM extract	na	na
Ace extract	3.39 ± 0.01 ^e^	4.8 ± 1.0 ^d^
MeOH extract	18.64 ± 0.00 ^b^	26.2 ± 0.2 ^b^
MeOH-H_2_O extract	16.95 ± 0.01 ^d^	23.8 ± 0.2 ^c^
H_2_O extract	22.03 ± 0.02 ^a^	28.6 ± 0.3 ^a^
Caperatic acid	18.25 ± 0.02 ^c^	25.9 ± 0.1 ^b^

Mean values within a column with the same letter are not significantly different at *p* 0.05 using Duncan’s test. The first letter of the alphabet for the highest values, the next for statistically significant decreasing values. The mean values ± SD from at least *n* = 4 independent measurements are presented; na—not active.

**Table 8:** The IC_50_ of the analysed lichen-derived extracts established after 48 h treatment of T98G and U-138 MG cell lines.

Extracts/ Substance	T98G	U-138 MG
	IC_50_ (µg/mL)	
DCM	65.8 ± 18.9 ^a^	72.8 ± 2.0 ^a^
Ace	44.8 ± 10.0 ^a^	65.7 ± 5.0 ^a^
MeOH	31.8 ± 10.4 ^a^	70.6 ± 11.9 ^a^
MeOH-H_2_O	57.8 ± 19.9 ^a^	IC_50_ 100 ^a^
H_2_O	46.8 ± 11.0 ^a^	88.2 ± 15.2 ^a^
Caperatic acid	100 *	100 *

Mean values within a column with the same letter are not significantly different at *p* 0.05 using Duncan’s test. The first letter of the alphabet for the highest values, the next for statistically significant decreasing values. IC_50_ ± SEM was calculated from the results obtained in three independent experiments with four measurements per assay, for each included in the dose-response curves assessed by the MTT assay or taken from the literature (*—[15]).

**Table 9:** The total biological potentials of *P. glauca* extracts as neuroprotective and Anti-GBM biological characteristic.

Extract	DCM	Ace	MeOH	MeOH-H_2_O	H_2_O
Neuroprotective potential [%]	59.74	60.02	60.66	54.57	41.78
Anti-GBM potential [%]	33.74	33.67	35.17	36.43	31.39

The presented values in every of two field (neuroprotective potential and anti-GBM potential) were summarized by adding the % of each individual feature, taking into account the number of variables.

## Data Availability

The data supporting reported results were be found in: Department of Pharmacognosy, Poznan University of Medical Sciences; Department of Pharmaceutical Biochemistry, Poznan University of Medical Sciences; Department of Biotechnology, Microbiology and Human Nutrition, University of Life Sciences in Lublin and Department of Clinical Genetics, Medical University of Lublin; Centre for Advanced Technologies, Adam Mickiewicz University in Poznań.

## Supplementary Materials

The following supporting information can be downloaded at: https://www.mdpi.com/article/10.3390/antiox11102069/s1, Table S1. Chemical shifts data of caperatic acid in acetone-d_6_ (^1^H-, ^13^C NMR; 700 MHz). Table S2. Selected characteristic bands of DCM, Ace, MeOH, MeOH-H_2_O, H_2_O extract, caperatic acid, atranorin and methyl β-orcinolcarboxylate.

## References

[1] Feigin V.L., Nichols E., Alam T., Bannick M.S., Beghi E., Blake N., Culpepper W.J., Dorsey E.R., Elbaz A., Ellenbogen R.G. (2019). Global, regional, and national burden of neurological disorders, 1990–2016: A systematic analysis for the Global Burden of Disease Study 2016. Lancet Neurol..

[2] Javaid S.F., Giebel C., Khan M.A.B., Hashim M.J. (2021). Epidemiology of Alzheimer’s disease and other dementias: Rising global burden and forecasted trends. F1000Research.

[3] Poddar M.K., Chakraborty A., Banerjee S. (2021). Neurodegeneration: Diagnosis, prevention, and therapy. Oxidoreductase.

[4] Teleanu R.I., Niculescu A.-G., Roza E., Vladâcenco O., Grumezescu A.M., Teleanu D.M. (2022). Neurotransmitters—Key Factors in Neurological and Neurodegenerative Disorders of the Central Nervous System. Int. J. Mol. Sci..

[5] Pritam P., Deka R., Bhardwaj A., Srivastava R., Kumar D., Jha A.K., Jha N.K., Villa C., Jha S.K. (2022). Antioxidants in Alzheimer’s disease: Current therapeutic significance and future prospects. Biology.

[6] Grech N., Dalli T., Mizzi S., Meilak L., Calleja N., Zrinzo A. (2020). Rising incidence of glioblastoma multiforme in a well-defined population. Cureus.

[7] Zhao T., Li C., Ge H., Lin Y., Kang D. (2022). Glioblastoma vaccine tumor therapy research progress. Chin. Neurosurg. J..

[8] Ogawa K., Kurose A., Kamataki A., Asano K., Katayama K., Kurotaki H. (2020). Giant cell glioblastoma is a distinctive subtype of glioma characterized by vulnerability to DNA damage. Brain Tumor Pathol..

[9] Tagde P., Tagde P., Tagde S., Bhattacharya T., Garg V., Akter R., Rahman M.H., Najda A., Albadrani G.M., Sayed A.A. (2021). Natural bioactive molecules: An alternative approach to the treatment and control of glioblastoma multiforme. Biomed. Pharmacother..

[10] Sharifi-Rad M., Lankatillake C., Dias D.A., Docea A.O., Mahomoodally M.F., Lobine D., Chazot P.L., Kurt B., Boyunegmez Tumer T., Catarina Moreira A. (2020). Impact of natural compounds on neurodegenerative disorders: From preclinical to pharmacotherapeutics. J. Clin. Med..

[11] Kubczak M., Szustka A., Rogalińska M. (2021). Molecular targets of natural compounds with anti-cancer properties. Int. J. Mol. Sci..

[12] Persano F., Gigli G., Leporatti S. (2022). Natural Compounds as Promising Adjuvant Agents in the Treatment of Gliomas. Int. J. Mol. Sci..

[13] Studzińska-Sroka E., Majchrzak-Celińska A., Zalewski P., Szwajgier D., Baranowska-Wójcik E., Kaproń B., Plech T., Żarowski M., Cielecka-Piontek J. (2021). Lichen-Derived Compounds and Extracts as Biologically Active Substances with Anticancer and Neuroprotective Properties. Pharmaceuticals.

[14] Studzińska-Sroka E., Majchrzak-Celińska A., Zalewski P., Szwajgier D., Baranowska-Wójcik E., Żarowski M., Plech T., Cielecka-Piontek J. (2021). Permeability of Hypogymnia physodes Extract Component—Physodic Acid through the Blood–Brain Barrier as an Important Argument for Its Anticancer and Neuroprotective Activity within the Central Nervous System. Cancers.

[15] Majchrzak-Celińska A., Kleszcz R., Studzińska-Sroka E., Łukaszyk A., Szoszkiewicz A., Stelcer E., Jopek K., Rucinski M., Cielecka-Piontek J., Krajka-Kuźniak V. (2022). Lichen Secondary Metabolites Inhibit the Wnt/β-Catenin Pathway in Glioblastoma Cells and Improve the Anticancer Effects of Temozolomide. Cells.

[16] Mitrovic T., Stamenkovic S., Cvetkovic V., Radulovic N., Mladenovic M., Stankovic M., Topuzovic M., Radojevic I., Stefanovic O., Vasic S. (2014). Platismatia glaucia and Pseudevernia furfuracea lichens as sources of antioxidant, antimicrobial and antibiofilm agents. EXCLI J..

[17] Ingelfinger R., Henke M., Roser L., Ulshöfer T., Calchera A., Singh G., Parnham M.J., Geisslinger G., Fürst R., Schmitt I. (2020). Unraveling the Pharmacological Potential of Lichen Extracts in the Context of Cancer and Inflammation With a Broad Screening Approach. Front. Pharmacol..

[18] Obermayer W., Randlane T. (2012). Morphological and chemical studies on Platismatia erosa (Parmeliaceae) from Tibet, Nepal and Bhutan. Bryologist.

[19] Abdallah E.M. (2019). Evaluation of antimicrobial activity of a lichen used as a spice (*Platismatia glauca*). Adv. Life Sci..

[20] Gulluce M., Aslan A., Sokmen M., Sahin F., Adiguzel A., Agar G., Sokmen A. (2006). Screening the antioxidant and antimicrobial properties of the lichens Parmelia saxatilis, Platismatia glauca, Ramalina pollinaria, Ramalina polymorpha and Umbilicaria nylanderiana. Phytomedicine.

[21] Šeklić D.S., Obradović A.D., Stanković M.S., Živanović M.N., Mitrović T.L., Stamenković S.M., Marković S.D. (2018). Proapoptotic and antimigratory effects of Pseudevernia furfuracea and Platismatia glauca on colon cancer cell lines. Food Technol. Biotechnol..

[22] Bézivin C., Tomasi S., Lohézic-Le Dévéhat F., Boustie J. (2003). Cytotoxic activity of some lichen extracts on murine and human cancer cell lines. Phytomedicine.

[23] Paluszczak J., Kleszcz R., Studzińska-Sroka E., Krajka-Kuźniak V. (2018). Lichen-derived caperatic acid and physodic acid inhibit Wnt signaling in colorectal cancer cells. Mol. Cell. Biochem..

[24] Chanaj-Kaczmarek J., Wysocki M., Karachitos A., Wojcińska M., Bartosz G., Matławska I., Kmita H. (2015). Effects of plant extract antioxidative phenolic compounds on energetic status and viability of Saccharomyces cerevisiae cells undergoing oxidative stress. J. Funct. Foods.

[25] Studzińska-Sroka E., Galanty A., Gościniak A., Wieczorek M., Kłaput M., Dudek-Makuch M., Cielecka-Piontek J. (2021). Herbal Infusions as a Valuable Functional Food. Nutrients.

[26] Santos J.S., Brizola V.R.A., Granato D. (2017). High-throughput assay comparison and standardization for metal chelating capacity screening: A proposal and application. Food Chem..

[27] Watanabe M., de Moura Neiva L.B., da Costa Santos C.X., Laurindo F.R.M., Vattimo M.d.F.F. (2007). Isoflavone and the heme oxygenase system in ischemic acute kidney injury in rats. Food Chem. Toxicol..

[28] Szutowicz A., Kobes R.D., Orsulak P.J. (1984). Colorimetric assay for monoamine oxidase in tissues using peroxidase and 2, 2′-azinodi (3-ethylbenzthiazoline-6-sulfonic acid) as chromogen. Anal. Biochem..

[29] Parschat K., Canne C., Hüttermann J., Kappl R., Fetzner S. (2001). Xanthine dehydrogenase from Pseudomonas putida 86: Specificity, oxidation–reduction potentials of its redox-active centers, and first EPR characterization. Biochim. Biophys. Acta (BBA)-Protein Struct. Mol. Enzymol..

[30] Moreira P.R., Maioli M.A., Medeiros H.C.D., Guelfi M., Pereira F.T.V., Mingatto F.E. (2014). Protective effect of bixin on carbon tetrachloride-induced hepatotoxicity in rats. Biol. Res..

[31] Singh R.P., Padmavathi B., Rao A.R. (2000). Modulatory influence of Adhatoda vesica (*Justicia adhatoda*) leaf extract on the enzymes of xenobiotic metabolism, antioxidant status and lipid peroxidation in mice. Mol. Cell. Biochem..

[32] Morris G.M., Huey R., Lindstrom W., Sanner M.F., Belew R.K., Goodsell D.S., Olson A.J. (2009). AutoDock4 and AutoDockTools4: Automated docking with selective receptor flexibility. J. Comput. Chem..

[33] RCSB Protein Data Bank. https://www.rcsb.org.

[34] PubChem. https://pubchem.ncbi.nlm.nih.gov/.

[35] Dennington R., Keith T.A., Millam J.M. (2016). GaussView.

[36] UCSF Chimera. https://www.cgl.ucsf.edu/chimera/.

[37] Pettersen E.F., Goddard T.D., Huang C.C., Couch G.S., Greenblatt D.M., Meng E.C., Ferrin T.E. (2004). UCSF Chimera—A visualization system for exploratory research and analysis. J. Comput. Chem..

[38] Open Babel Program. http://openbabel.org.

[39] O’Boyle N.M., Banck M., James C.A., Morley C., Vandermeersch T., Hutchison G.R. (2011). Open Babel: An open chemical toolbox. J. Cheminform..

[40] Adasme M.F., Linnemann K.L., Bolz S.N., Kaiser F., Salentin S., Haupt V.J., Schroeder M. (2021). PLIP 2021: Expanding the scope of the protein–ligand interaction profiler to DNA and RNA. Nucleic Acids Res..

[41] Protein-Ligand Interaction Profiler. https://plip-tool.biotec.tu-dresden.de/.

[42] Support|pymol.org. https://pymol.org/2/support.html?.

[43] Majchrzak-Celińska A., Zielińska-Przyjemska M., Wierzchowski M., Kleszcz R., Studzińska-Sroka E., Kaczmarek M., Paluszczak J., Cielecka-Piontek J., Krajka-Kuźniak V. (2021). Methoxy-stilbenes downregulate the transcription of Wnt/β-catenin-dependent genes and lead to cell cycle arrest and apoptosis in human T98G glioblastoma cells. Adv. Med. Sci..

[44] Chanaj-Kaczmarek J., Rosiak N., Szymanowska D., Rajewski M., Wender-Ozegowska E., Cielecka-Piontek J. (2022). The Chitosan-Based System with Scutellariae baicalensis radix Extract for the Local Treatment of Vaginal Infections. Pharmaceutics.

[45] DeNoyer L.K., Dodd J.G. (2006). Smoothing and derivatives in spectroscopy. Handbook of Vibrational Spectroscopy.

[46] Xu C.-H., Liu S.-L., Zhao S.-N., Li S.-Z., Sun S.-Q. (2013). Unveiling ontogenesis of herbal medicine in plant chemical profiles by infrared macro-fingerprinting. Planta Med..

[47] Apak R., Güçlü K., Özyürek M., Çelik S.E. (2008). Mechanism of antioxidant capacity assays and the CUPRAC (cupric ion reducing antioxidant capacity) assay. Microchim. Acta.

[48] Ruiz J.C.R., Ordoñez Y.B.M., Basto Á.M., Campos M.R.S. (2015). Antioxidant capacity of leaf extracts from two Stevia rebaudiana Bertoni varieties adapted to cultivation in Mexico. Nutr. Hosp..

[49] Winiarska-Mieczan A., Baranowska-Wójcik E., Kwiecień M., Grela E.R., Szwajgier D., Kwiatkowska K., Kiczorowska B. (2020). The role of dietary antioxidants in the pathogenesis of neurodegenerative diseases and their impact on cerebral oxidoreductive balance. Nutrients.

[50] Sil S., Ghosh T. (2016). Role of cox-2 mediated neuroinflammation on the neurodegeneration and cognitive impairments in colchicine induced rat model of Alzheimer’s disease. J. Neuroimmunol..

[51] Soria F.N., Paviolo C., Doudnikoff E., Arotcarena M.-L., Lee A., Danné N., Mandal A.K., Gosset P., Dehay B., Groc L. (2020). Synucleinopathy alters nanoscale organization and diffusion in the brain extracellular space through hyaluronan remodeling. Nat. Commun..

[52] Jin S.-G., Jeong Y.-I., Jung S., Ryu H.-H., Jin Y.-H., Kim I.-Y. (2009). The effect of hyaluronic acid on the invasiveness of malignant glioma cells: Comparison of invasion potential at hyaluronic acid hydrogel and matrigel. J. Korean Neurosurg. Soc..

[53] Ferrer V.P., Moura Neto V., Mentlein R. (2018). Glioma infiltration and extracellular matrix: Key players and modulators. Glia.

[54] Millot M., Tomasi S., Articus K., Rouaud I., Bernard A., Boustie J. (2007). Metabolites from the lichen Ochrolechia parella growing under two different heliotropic conditions. J. Nat. Prod..

[55] Neupane B.P., Malla K.P., Gautam A., Chaudhary D., Paudel S., Timsina S., Jamarkattel N. (2017). Elevational trends in usnic acid concentration of lichen Parmelia flexilis in relation to temperature and precipitation. Climate.

[56] Chen Y., Qin C., Huang J., Tang X., Liu C., Huang K., Xu J., Guo G., Tong A., Zhou L. (2020). The role of astrocytes in oxidative stress of central nervous system: A mixed blessing. Cell Prolif..

[57] Marante F.J.T., Castellano A.G., Rosas F.E., Aguiar J.Q., Barrera J.B. (2003). Identification and quantitation of allelochemicals from the lichen Lethariella canariensis: Phytotoxicity and antioxidative activity. J. Chem. Ecol..

[58] Fernández-Moriano C., Gómez-Serranillos M.P., Crespo A. (2016). Antioxidant potential of lichen species and their secondary metabolites. A systematic review. Pharm. Biol..

[59] Kontoghiorghes G.J., Kontoghiorghe C.N. (2020). Iron and Chelation in Biochemistry and Medicine: New Approaches to Controlling Iron Metabolism and Treating Related Diseases. Cells.

[60] Baldari S., Di Rocco G., Toietta G. (2020). Current Biomedical Use of Copper Chelation Therapy. Int. J. Mol. Sci..

[61] Leuci R., Brunetti L., Laghezza A., Loiodice F., Tortorella P., Piemontese L. (2020). Importance of biometals as targets in medicinal chemistry: An overview about the role of Zinc (II) chelating agents. Appl. Sci..

[62] Bukhari S.N.A. (2022). Dietary Polyphenols as Therapeutic Intervention for Alzheimer’s Disease: A Mechanistic Insight. Antioxidants.

[63] Suno H., Machida M., Dohi T., Ohmura Y. (2021). Quantum chemical calculation studies toward microscopic understanding of retention mechanism of Cs radioisotopes and other alkali metals in lichens. Sci. Rep..

[64] Maslać A., Maslać M., Tkalec M. (2016). The impact of cadmium on photosynthetic performance and secondary metabolites in the lichens Parmelia sulcata, Flavoparmelia caperata and Evernia prunastri. Acta Bot. Croat..

[65] Purvis O.W. (2014). Adaptation and interaction of saxicolous crustose lichens with metals. Bot. Stud..

[66] Hauck M., Huneck S. (2007). Lichen substances affect metal adsorption in Hypogymnia physodes. J. Chem. Ecol..

[67] Hauck M. (2008). Metal homeostasis in Hypogymnia physodes is controlled by lichen substances. Environ. Pollut..

[68] Hauck M., Willenbruch K., Leuschner C. (2009). Lichen substances prevent lichens from nutrient deficiency. J. Chem. Ecol..

[69] Denoyer D., Masaldan S., La Fontaine S., Cater M.A. (2015). Targeting copper in cancer therapy:‘Copper That Cancer’. Metallomics.

[70] Zalewska-Ziob M., Adamek B., Kasperczyk J., Romuk E., Hudziec E. (2019). Chwali nska, E.; Dobija-Kubica, K.; Rogozinski, P.; Brulinski, K. Activity of Antioxidant Enzymes in the Tumor and Adjacent Noncancerous Tissues of Non-Small-Cell Lung Cancer. Oxid. Med. Cell. Longev..

[71] Backos D.S., Franklin C.C., Reigan P. (2012). The role of glutathione in brain tumor drug resistance. Biochem. Pharmacol..

[72] Guo J., Zhao X., Li Y., Li G., Liu X. (2018). Damage to dopaminergic neurons by oxidative stress in Parkinson’s disease. Int. J. Mol. Med..

[73] Pu P., Lan J., Shan S., Huang E., Bai Y., Guo Y., Jiang D. (1996). Study of the antioxidant enzymes in human brain tumors. J. Neurooncol..

[74] Olivier C., Oliver L., Lalier L., Vallette F.M. (2021). Drug resistance in glioblastoma: The two faces of oxidative stress. Front. Mol. Biosci..

[75] Sharma K. (2019). Cholinesterase inhibitors as Alzheimer’s therapeutics. Mol. Med. Rep..

[76] Ma Y., Gao W., Ma S., Liu Y., Lin W. (2020). Observation of the elevation of cholinesterase activity in brain glioma by a near-infrared emission chemsensor. Anal. Chem..

[77] Thompson E.G., Sontheimer H. (2019). Acetylcholine receptor activation as a modulator of glioblastoma invasion. Cells.

[78] Coelho dos Santos T., Gomes T.M., Pinto B.A.S., Camara A.L., Antonio de Andrade Paes M. (2018). Naturally Occurring Acetylcholinesterase Inhibitors and Their Potential Use for Alzheimer’s Disease. Ther. Front. Pharmacol..

[79] Ahmed F., Ghalib R.M., Sasikala P., Ahmed K.K.M. (2013). Cholinesterase inhibitors from botanicals. Pharmacogn. Rev..

[80] Reddy R.G., Veeraval L., Maitra S., Chollet-Krugler M., Tomasi S., Lohezic-Le Devehat F., Boustie J., Chakravarty S. (2016). Lichen-derived compounds show potential for central nervous system therapeutics. Phytomedicine.

[81] Desai S.J., Prickril B., Rasooly A. (2018). Mechanisms of phytonutrient modulation of cyclooxygenase-2 (COX-2) and inflammation related to cancer. Nutr. Cancer.

[82] Zhou J.-R., Kanda Y., Tanaka A., Manabe H., Nohara T., Yokomizo K. (2016). Anti-hyaluronidase activity in vitro and amelioration of mouse experimental dermatitis by tomato saponin, Esculeoside A. J. Agric. Food Chem..

[83] Dharmajaya R., Sari D.K. (2021). Role and value of inflammatory markers in brain tumors: A case controlled study. Ann. Med. Surg..

[84] Monslow J., Govindaraju P., Puré E. (2015). Hyaluronan—A functional and structural sweet spot in the tissue microenvironment. Front. Immunol..

[85] Majchrzak-Celińska A., Misiorek J.O., Kruhlenia N., Przybyl L., Kleszcz R., Rolle K., Krajka-Kuźniak V. (2021). COXIBs and 2,5-dimethylcelecoxib counteract the hyperactivated Wnt/β-catenin pathway and COX-2/PGE2/EP4 signaling in glioblastoma cells. BMC Cancer.

[86] Šeklić D.S., Jovanović M.M. (2022). Platismatia glauca—Lichen species with suppressive properties on migration and invasiveness of two different colorectal carcinoma cell lines. J. Food Biochem..

