# Supplementary material for: Is Caperatic Acid the Only Compound Responsible for Activity of Lichen Platismatia glauca within the Nervous System?
Source: Antioxidants (Basel). 2022 Oct 20;11(10):2069. doi: 10.3390/antiox11102069 (PMC9598164; doi:10.3390/antiox11102069)
Supplement: Supplementary file 1 [file antioxidants-11-02069-s001.zip › antioxidants-1928385-supplementary.pdf]

antioxidants

Supplementary Materials: Is caperatic acid the only compound responsible for activity of lichen *Platismatia glauca* within the nervous system?

Elżbieta Studzińska-Sroka, Aleksandra Majchrzak-Celińska, Monika Bańdurska, Natalia Rosiak, Dominik Szwajgier, Ewa Baranowska-Wójcik, Marcin Szymański, Wojciech Gruszka and Judyta Cielecka-Piontek

Table S1. Chemical shifts data of caperatic acid in acetone-d_6 (^{1}H-, ^{13}C NMR; 700 MHz).

Position	Caperatic acid			
	δ C(ppm)	δ H(ppm)	multiplicity, *J* in Hz	
1	169.93	-	-	-
2	40.32	3.10	d (16.1)	2H
		2.76	d (16.8)	
3	74.85	-	-	-
4	52.27	2.67	dd (11.9, 2.8)	H
5-17	21.90-31.26	1.30	bs	26H
18	12.98	0.86	t	3H
19	173.43	-	-	-
20	172.49	-	-	-
21	50.45	3.60	s	3H

d - doublet; dd - doublet of doublet; s - singlet; bs - broad singlet; t – triplet. ^{1}H- and ^{13}C-NMR spectra were recorded at 700 MHz on a Bruker AVANCE III 700 NMR spectrometer. Data was comparable to the published values (Huneck & Yoshimura 1996).

Table S2. Selected characteristic bands of DCM, Ace, MeOH, MeOH-H_2O, H_2O extract, caperatic acid, atranorin and methyl β-orcinolcarboxylate.

	Extract	Caperatic acid	Atranorin	Methyl β-orcinolcarboxylate
DCM extract	536	536		
	795		795	
	808		808	
	941	941		
	1028			1028
	1074		1070	
	1113	1113		
	1155			1155
	1369	1369		

	1410	1410		
	1447	1447		
	1576		1576	
	1620		1620	
	1682	1682		
	1740	1740		
	2849	2849		
	2884	2884		
	2916	2916		
	2957	2957		
Ace extract	534	534		
	642	642		
	721	721		
	795	795		
	853	853		
	941	941		
	1078		1070	
	1111		1107	
	1153		1161	
	1182	1182		
	1250	1250		
	1261		1260	
	1287	1287		
	1304	1304		
	1369	1369		
	1410	1410		
	1447	1447		
	1578		1574	
	1618		1620	
	1647		1653	
	1686	1686		
	1738	1738		
	2849	2849		
	2884	2884		
	2914	2914		
	2957	2957		
MeOH extract	640	640		
	862		862	
	937			937
	986			986
	1207		1204	
	1368			1366

	1410	1410	
	1445	1445	
	1618		1620
	1651		1653
	1686	1684	
	1734	1740	
	2849	2849	
	2884	2884	
	2918	2918	
MeOH-H_2O extract	1250	1250	
	1368	1369	
	1408	1410	
	1682	1684	
	1734	1738	
	2849	2849	
	2884	2884	
	2916	2916	
H_2O extract	870	856	
	1082	1082	
	1404		
	1672	1672	
	1724	1724	
